# Global epidemiology of campylobacteriosis and the impact of COVID-19

**DOI:** 10.3389/fcimb.2022.979055

**Published:** 2022-11-28

**Authors:** Fang Liu, Seul A. Lee, Jessica Xue, Stephen M. Riordan, Li Zhang

**Affiliations:** ^1^ School of Biotechnology and Biomolecular Sciences, University of New South Wales, Sydney, NSW, Australia; ^2^ Faculty of Medicine, Monash University, Melbourne, VIC, Australia; ^3^ Gastrointestinal and Liver Unit, Prince of Wales Hospital, University of New South Wales, Sydney, NSW, Australia

**Keywords:** campylobacteriosis, *Campylobacter*, *Campylobacter jejuni*, epidemiology, diarrhea, gastroenteritis, transmission

## Abstract

Campylobacteriosis is a gastroenteritis caused by pathogenic *Campylobacter* species and an important topic in public health. Here we review the global epidemiology of campylobacteriosis in the last eight years between 2014-2021, providing comprehensive and updated information on the reported incidence and outbreaks of *Campylobacter* infections. The government public health website of each of the 195 countries and publications from 2014 to September 2022 in public databases were searched. The reported incidence of campylobacteriosis in pre-COVID-19 years was compared to that during the COVID-19 pandemic in countries where data were available. Czech Republic had the highest reported incidence of campylobacteriosis worldwide (215 per 100,000 in 2019), followed by Australia (146.8 per 100,000 in 2016) and New Zealand (126.1 per 100,000 in 2019). *Campylobacter* was one of the most common human enteric pathogens in both developed and developing countries. About 90% of cases of campylobacteriosis were caused by *Campylobacter jejuni*, whereas less than 10% of cases were caused by *Campylobacter coli*. Other *Campylobacter* species were also isolated. The reported incidence and case numbers of campylobacteriosis in developed nations have remained steadily high prior to the COVID-19 pandemic, whilst some countries reported an increasing trend such as France and Japan. While outbreaks were more frequently reported in some countries, *Campylobacter* infections were mainly sporadic cases in most of the developed countries. *Campylobacter* infection was more common in summer in some but not all countries. *Campylobacter* infection was more common in males than females. The COVID-19 pandemic has reduced the reported incidence of campylobacteriosis in most countries where 2020 epidemiology data were available. In conclusion, *Campylobacter* infection remains a global health concern. Increased research and improved strategies are needed for prevention and reduction of *Campylobacter* infection.

## 1 Introduction

Campylobacteriosis is a gastroenteritis caused by pathogenic *Campylobacter* species, which are gram-negative bacteria with a curved or spiral shape. Bacterial cells of most *Campylobacter* species are motile with a flagellum present at one or both ends of the bacteria, allowing them to have a corkscrew-like motion during movement (Lastovica et al., 2014). Cases of campylobacteriosis in humans are mainly caused by *Campylobacter jejuni* and most commonly present as gastroenteritis. Individuals suffering from *Campylobacter* infection often have diarrhea, fever, and abdominal pain; sometimes nausea and vomiting are also present. Illness usually resolves within two to five days but may last up to several weeks. It is usually self-limiting, with 5-10% requiring hospitalisation and a fatality rate of five in 10,000 (Galanis, 2007). In a very small number of cases (one in every 1,000), *C. jejuni* also causes Guillain-Barré syndrome, an autoimmune neurological disease triggered by the molecular mimicry between *C. jejuni* outer membrane lipooligosaccharides and human peripheral nerve gangliosides (Li et al., 2020a; Centers for Disease Control and Prevention, 2022). Campylobacteriosis is most often caused by consumption of contaminated poultry; ruminants such as cattle, sheep and goats can also be a source for human *Campylobacter* infection (Chlebicz and Śliżewska, 2018). Several *Campylobacter* species use humans as their natural host and are referred to as human hosted *Campylobacter* species (Liu et al., 2018). Translocation of some of the human hosted *Campylobacter* species such as *Campylobacter concisus* may cause chronic inflammatory diseases of the gastrointestinal tract (Liu et al., 2018).

*Campylobacter* is a common gastroenteric bacterial pathogen in humans. Given this, the global epidemiology of campylobacteriosis is an important topic in public health. Here we review the global epidemiology of campylobacteriosis in the last eight years between 2014-2021, providing comprehensive and updated information on the reported incidence and outbreaks of *Campylobacter* infections. The reported incidence of campylobacteriosis in pre-COVID-19 years was compared to that during the COVID-19 pandemic within individual countries where data were available.

## 2 Data collection

The government public health website of each of the 195 countries was searched. Data that were available in English were directly used in this review. In countries where data were presented in non-English language, the reported campylobacteriosis incidence and case numbers were obtained from English publications that were based on their government data.

Furthermore, publications on human *Campylobacter* infection using data collected from 2014 to September 2022 in PubMed and Web of Science were searched. Keyword combinations of country or continent name with *Campylobacter*, campylobacteriosis, diarrhea, enteritis, or gastroenteritis were used. The search yielded 4,766 publications. Examination of these 4,766 publications revealed that 66 publications contained data of *Campylobacter* outbreaks and sporadic cases between 2014 and 2021, which were included in this review. Other relevant publications were included in the discussion.

## 3 Epidemiology

### 3.1 Reported incidence and outbreaks of campylobacteriosis in countries with national surveillance data

Campylobacteriosis is a notifiable disease in countries with surveillance programs available (**Table 1**). Most of the countries with surveillance programs provided data of both incidence and case numbers, while some only provided case numbers. The reported incidence and case numbers of campylobacteriosis in countries with surveillance programs are in **Table 1**, arranged alphabetically based on continents and countries within each continent.

**Table 1:** Incidence and national case numbers of campylobacteriosis between 2014-2021 in countries with national surveillance.

Country	Year	Incidence per 100,000	Total no. of cases	*C. jejuni*	*C. coli*	Other *Campylobacter* species	Age (yr) (% of cases or incidence per 100,000)	Gender (M:F or incidence per 100,000)	Season^#^	Reference
**Asia**										
Japan	2018		1995^#^						Summer	(Vetchapitak and Misawa, 2019)
	2017		2315^#^						Summer	(Vetchapitak and Misawa, 2019)
	2016		3272^#^						Summer	(Vetchapitak and Misawa, 2019)
	2015		2089^#^						Summer	(Vetchapitak and Misawa, 2019)
	2014		1893^#^						Summer	(Vetchapitak and Misawa, 2019)
Korea	2019		270^#^						Summer	(Korea Disease Control and Prevention Agency, 2021)
	2018		542^#^						Summer	(Korea Disease Control and Prevention Agency, 2019)
	2017		103^#^						Summer	(Korea Disease Control and Prevention Agency, 2018)
	2016		902^#^						Summer	(Korea Disease Control and Prevention Agency, 2017)
	2015		747^#^						Summer	(Korea Disease Control and Prevention Agency, 2016)
	2014		619^#^						Summer	(Korea Disease Control and Prevention Agency, 2015)
Singapore	2018	7.6	427	371/427	40/427	16/427	0-4 (71.7)	1.5:1		(Ministry of Health Singapore, 2019)
	2017	8.8	495	379/475	40/475	76/475	0-4 (64.5)	1.2:1		(Ministry of Health Singapore, 2018)
	2016	7.6	442	364/442	33/442	45/442	0-4 (64.4)	1.1:1		(Ministry of Health Singapore, 2017)
	2015	7.6	420	334/420	31/420	55/420	0-4 (76.4)	1:1.4		(Ministry of Health Singapore, 2016)
	2014	8.0	435	370/435	18/435	45/435	0-4 (72.5)	1.3:1		(Ministry of Health Singapore, 2015)
**Europe**										
Europe	2020	40.4	120,946	88.1%^	10.6%^	1.3%^			Summer	(European Food Safety Authority, 2021b)
	2019	59.7	220.682	83.1%^	10.8%^	6.1%^			Summer	(European Food Safety Authority, 2021a)
	2018	64.1	246,571	83.9%^	10.3%^	5.8%^			Summer	(European Food Safety Authority, 2019a)
	2017	64.9	246,194				<5 (13.4%; 138.0)	1.2:1	Summer	(European Centre for Disease Prevention and Control, 2019; European Food Safety Authority, 2019a)
	2016	66.4	246,980				<5 (13.4%; 144.4)	1.2:1	Summer	(European Centre for Disease Prevention and Control, 2018b; European Food Safety Authority, 2019a)
	2015	63.0	232,226				<5 (13.0%; 181.2)	1.2:1	Summer	(European Centre for Disease Prevention and Control, 2018a; European Food Safety Authority, 2019a)
	2014	66.5	236,818							(European Centre for Disease Prevention and Control, 2018a)
Austria	2020	60.7	5,406							(European Food Safety Authority, 2021b)
	2019	74.2	6,573							(European Food Safety Authority, 2021a)
	2018	90.7	7,999							(Austrian Agency for Health and Food Safety, 2020)
	2017	82.1	7,204							(European Centre for Disease Prevention and Control, 2019)
	2016	81.4	7,083							(European Centre for Disease Prevention and Control, 2019)
	2015	72.9	6,258							(European Centre for Disease Prevention and Control, 2019)
	2014	76.6	6,514							(European Centre for Disease Prevention and Control, 2019)
Belgium	2020	48.6	5,595							(European Food Safety Authority, 2021b)
	2019	64.0	7,337							(European Food Safety Authority, 2021a)
	2018	70.9	8,086							(European Food Safety Authority, 2021a)
	2017	76.2	8,649							(European Centre for Disease Prevention and Control, 2019)
	2016	88.9	10,055							(European Centre for Disease Prevention and Control, 2019)
	2015	80.7	9,066							(European Centre for Disease Prevention and Control, 2019)
	2014		8,098							(European Centre for Disease Prevention and Control, 2019)
Bulgaria	2020	1.8	127							
	2019	3.3	229							(European Food Safety Authority, 2021a)
	2018	2.7	191							(European Food Safety Authority, 2021a)
	2017	2.7	195							(European Centre for Disease Prevention and Control, 2019)
	2016	2.8	202							(European Centre for Disease Prevention and Control, 2019)
	2015	3.2	227							(European Centre for Disease Prevention and Control, 2019)
	2014	2.0	144							(European Centre for Disease Prevention and Control, 2019)
Croatia	2020	26.0	1,054							(European Food Safety Authority, 2021b)
	2019	42.2	1,722							(European Food Safety Authority, 2021a)
	2018	47.9	1,965							(European Food Safety Authority, 2021a)
	2017	40.6	1,686							(European Centre for Disease Prevention and Control, 2019)
	2016	36.4	1,524							(European Centre for Disease Prevention and Control, 2019)
	2015	33.0	1,393							(European Centre for Disease Prevention and Control, 2019)
	2014	38.8	1,647							(European Centre for Disease Prevention and Control, 2019)
Cyprus	2020	2.0	18							(European Food Safety Authority, 2021b)
	2019	2.4	21							(European Food Safety Authority, 2021a)
	2018	3.0	26							(European Food Safety Authority, 2021a)
	2017	2.3	20							(European Centre for Disease Prevention and Control, 2019)
	2016	2.5	21							(European Centre for Disease Prevention and Control, 2019)
	2015	3.4	29							(European Centre for Disease Prevention and Control, 2019)
	2014	4.7	40							(European Centre for Disease Prevention and Control, 2019)
Czech Republic	2020	163.8	17,517							(European Food Safety Authority, 2021b)
	2019	215.0	22,894							(European Food Safety Authority, 2021a)
	2018	215.8	22,895							(European Food Safety Authority, 2021a)
	2017	230.0	24,326							(European Centre for Disease Prevention and Control, 2019)
	2016	228.2	24,084							(European Centre for Disease Prevention and Control, 2019)
	2015	198.9	20,960							(European Centre for Disease Prevention and Control, 2019)
	2014	197.4	20,750							(European Centre for Disease Prevention and Control, 2019)
Denmark	2020	64.3	3,742							(European Food Safety Authority, 2021b)
	2019	93	5,389	652/701^	49/701^		>85	1.2:1		(Statens Serum Institut, 2019; Joensen et al., 2021)
	2018	79	4,547					1.1:1		(Statens Serum Institut, 2019)
	2017	74	4,243	408/435^	27/435^		20-29	1.1:1		(Statens Serum Institut, 2018)
	2016	82	4,678					1.2:1		(Statens Serum Institut, 2018)
	2015	76.5	4,327							(European Centre for Disease Prevention and Control, 2019)
	2014	67.0	3,773							(European Centre for Disease Prevention and Control, 2019)
Estonia	2020	19.9	265							(European Food Safety Authority, 2021b)
	2019	26.2	347							(European Food Safety Authority, 2021a)
	2018	31.2	411							(European Food Safety Authority, 2021a)
	2017	21.7	285							(European Centre for Disease Prevention and Control, 2019)
	2016	22.6	298							(European Centre for Disease Prevention and Control, 2019)
	2015	24.2	318							(European Centre for Disease Prevention and Control, 2019)
	2014	21.7	285							(European Centre for Disease Prevention and Control, 2019)
Finland	2020	37.5	2,074							(European Food Safety Authority, 2021b)
	2019	79.4	4,382							(European Food Safety Authority, 2021a)
	2018	92.5	5,099							(European Food Safety Authority, 2021a)
	2017	77.9	4,289							(European Centre for Disease Prevention and Control, 2019)
	2016	84.5	4,637							(European Centre for Disease Prevention and Control, 2019)
	2015	83.8	4,588							(European Centre for Disease Prevention and Control, 2019)
	2014	89.7	4,889							(European Centre for Disease Prevention and Control, 2019)
France	2020	58.8	7,920							(European Food Safety Authority, 2021b)
	2019	57.5	7,712							(European Food Safety Authority, 2021a)
	2018	56.0	7,491							(European Food Safety Authority, 2021a)
	2017	49.1	6,579							(European Centre for Disease Prevention and Control, 2019)
	2016	50.2	6,698							(European Centre for Disease Prevention and Control, 2019)
	2015	45.7	6,074							(European Centre for Disease Prevention and Control, 2019)
	2014	45.2	5,958							(European Centre for Disease Prevention and Control, 2019)
Germany	2020	55.8	46,379							(European Food Safety Authority, 2021b)
	2019	73.8	61,254							(European Food Safety Authority, 2021a)
	2018	81.6	67,585							(European Food Safety Authority, 2021a)
	2017	83.8	69,178							(European Centre for Disease Prevention and Control, 2019)
	2016	89.6	73,663							(European Centre for Disease Prevention and Control, 2019)
	2015	86.0	69,829							(European Centre for Disease Prevention and Control, 2019)
	2014	87.4	70,571							(European Centre for Disease Prevention and Control, 2019)
Greece	2020	2.0	218							(European Food Safety Authority, 2021b)
	2019	3.4	366							(European Food Safety Authority, 2021a)
	2018	3.3	357							(European Food Safety Authority, 2021a)
Hungary	2020	45.7	4,461							(European Food Safety Authority, 2021b)
	2019	65.5	6,400							(European Food Safety Authority, 2021a)
	2018	72.8	7,117							(European Food Safety Authority, 2021a)
	2017	79.7	7,807							(European Centre for Disease Prevention and Control, 2019)
	2016	87.0	8,556							(European Centre for Disease Prevention and Control, 2019)
	2015	84.6	8,342							(European Centre for Disease Prevention and Control, 2019)
	2014	85.5	8,444							(European Centre for Disease Prevention and Control, 2019)
Iceland	2020	26.1	95							(European Food Safety Authority, 2021b)
	2019	38.1	136							(European Food Safety Authority, 2021a)
	2018	41.6	145							(European Food Safety Authority, 2021a)
	2017	35.2	119							(European Centre for Disease Prevention and Control, 2019)
	2016	38.5	128							(European Centre for Disease Prevention and Control, 2019)
	2015	36.2	119							(European Centre for Disease Prevention and Control, 2019)
	2014	43.6	142							(European Centre for Disease Prevention and Control, 2019)
Ireland	2021		3,154							(Health Protection Surveillance Centre, 2022)
	2020	48.7	2,419							(European Food Safety Authority, 2021b; Health Protection Surveillance Centre, 2021)
	2019	56.6	2,777	222/257^	30/257^	5/257^				(Brehony et al., 2021; European Food Safety Authority, 2021a; Health Protection Surveillance Centre, 2021)
	2018	63.6	3,030	374/407^	30/407^	3/407^	0-4		Early summer	(Health Protection Surveillance Centre, 2019)
	2017	58.5	2,786	390/419^	26/419^	3/419^	0-4		Early summer	(Health Protection Surveillance Centre, 2018)
	2016	52.8	2,513	420/451^	27/451^		0-4		Early summer	(HSE Health Protection Surveillance Centre, 2018)
	2015	53.4	2,451	465/518^	53/518^				Early summer	(Health Protection Surveillance Centre, 2016)
	2014	55.9	2,593							(European Centre for Disease Prevention and Control, 2019)
Italy	2020		1,418							(European Food Safety Authority, 2021b)
	2019		1,633							(European Food Safety Authority, 2021a)
	2018		1,356							(European Food Safety Authority, 2021a)
	2017		1,060							(European Centre for Disease Prevention and Control, 2019)
	2016		1,057							(European Centre for Disease Prevention and Control, 2019)
	2015		1,014							(European Centre for Disease Prevention and Control, 2019)
	2014		1,252							(European Centre for Disease Prevention and Control, 2019)
Latvia	2020	5.5	104							(European Food Safety Authority, 2021b)
	2019	6.9	133							(European Food Safety Authority, 2021a)
	2018	4.5	87							(European Food Safety Authority, 2021a)
	2017	3.0	59							(European Centre for Disease Prevention and Control, 2019)
	2016	4.6	90							(European Centre for Disease Prevention and Control, 2019)
	2015	3.7	74							(European Centre for Disease Prevention and Control, 2019)
	2014	1.8	37							(European Centre for Disease Prevention and Control, 2019)
Lithuania	2020	42.3	1,183							(European Food Safety Authority, 2021b)
	2019	43.7	1,221							(European Food Safety Authority, 2021a)
	2018	32.7	919							(European Food Safety Authority, 2021a)
	2017	34.8	990							(European Centre for Disease Prevention and Control, 2019)
	2016	42.4	1,225							(European Centre for Disease Prevention and Control, 2019)
	2015	40.6	1,186							(European Centre for Disease Prevention and Control, 2019)
	2014	40.2	1,184							(European Centre for Disease Prevention and Control, 2019)
Luxembourg	2020	116.4	729							(European Food Safety Authority, 2021b)
	2019	44.1	271							(European Food Safety Authority, 2021a)
	2018	103.8	625							(European Food Safety Authority, 2021a)
	2017	103.8	613							(European Centre for Disease Prevention and Control, 2019)
	2016	89.9	518							(European Centre for Disease Prevention and Control, 2019)
	2015	45.1	254							(European Centre for Disease Prevention and Control, 2019)
	2014	158.8	873							(European Centre for Disease Prevention and Control, 2019)
Malta	2020	40.0	206							(European Food Safety Authority, 2021b)
	2019	56.3	278							(European Food Safety Authority, 2021a)
	2018	70.0	333							(European Food Safety Authority, 2021a)
	2017	50.2	231							(European Centre for Disease Prevention and Control, 2019)
	2016	47.1	212							(European Centre for Disease Prevention and Control, 2019)
	2015	56.4	248							(European Centre for Disease Prevention and Control, 2019)
	2014	67.1	288							(European Centre for Disease Prevention and Control, 2019)
Netherlands	2020	25.2	2,549							(European Food Safety Authority, 2021b)
	2019	34.1	3,415							(European Food Safety Authority, 2021a)
	2018	34.6	3,091							(European Food Safety Authority, 2021a)
	2017	32.5	2,890							(European Centre for Disease Prevention and Control, 2019)
	2016	38.3	3,383							(European Centre for Disease Prevention and Control, 2019)
	2015	43.0	3,778							(European Centre for Disease Prevention and Control, 2019)
	2014	47.5	4,159							(European Centre for Disease Prevention and Control, 2019)
Norway	2020	45.1	2,422							(European Food Safety Authority, 2021b)
	2019	78.0	4,154							(European Food Safety Authority, 2021a)
	2018	69.3	3,668							(European Food Safety Authority, 2021a)
	2017	73.9	3,884							(European Centre for Disease Prevention and Control, 2019)
	2016	44.5	2,317							(European Centre for Disease Prevention and Control, 2019)
	2015	44.9	2,318							(European Centre for Disease Prevention and Control, 2019)
	2014	66.3	3,386							(European Centre for Disease Prevention and Control, 2019)
Poland	2020	1.1	414							(European Food Safety Authority, 2021b)
	2019	1.9	715							(European Food Safety Authority, 2021a)
	2018	1.9	719							(European Food Safety Authority, 2021a)
	2017	2.3	874					1.3:1		(European Centre for Disease Prevention and Control, 2019; Radziszewski et al., 2019)
	2016	2.0	773	608/653^	45/653^			1.3:1		(Radziszewski et al., 2018; European Centre for Disease Prevention and Control, 2019)
	2015	1.7	653	511/532^	21/532^	1/532^		1.2:1		(Radziszewski et al., 2018; European Centre for Disease Prevention and Control, 2019)
	2014	1.7	650							(Sadkowska-Todys and Kucharczyk, 2016; European Centre for Disease Prevention and Control, 2019)
Portugal	2020	7.7	790							(European Food Safety Authority, 2021b)
	2019	8.6	887							(European Food Safety Authority, 2021a)
	2018	5.9	610							(European Food Safety Authority, 2021a)
	2017	5.8	596							(European Food Safety Authority, 2021a)
	2016	3.5	359							(European Food Safety Authority, 2021a)
	2015	2.6	271							(European Food Safety Authority, 2021a)
Romania	2020	1.6	300							(European Food Safety Authority, 2021b)
	2019	4.1	805							(European Food Safety Authority, 2021a)
	2018	2.9	573							(European Food Safety Authority, 2021a)
	2017	2.4	467							(European Centre for Disease Prevention and Control, 2019)
	2016	2.6	517							(European Centre for Disease Prevention and Control, 2019)
	2015	1.6	311							(European Centre for Disease Prevention and Control, 2019)
	2014	1.3	256							(European Centre for Disease Prevention and Control, 2019)
Slovakia	2020	90.2	4,921							(European Food Safety Authority, 2021b)
	2019	141.1	7,690							(European Food Safety Authority, 2021a)
	2018	153.2	8,339							(European Food Safety Authority, 2021a)
	2017	127.8	6,946							(European Centre for Disease Prevention and Control, 2019)
	2016	140.5	7,623							(European Centre for Disease Prevention and Control, 2019)
	2015	128.2	6,949							(European Centre for Disease Prevention and Control, 2019)
	2014	124.5	6,744							(European Centre for Disease Prevention and Control, 2019)
Slovenia	2020	38.7	811							(European Food Safety Authority, 2021b)
	2019	52.1	1,085							(European Food Safety Authority, 2021a)
	2018	63.1	1,305							(European Food Safety Authority, 2021a)
	2017	68.2	1,408							(European Centre for Disease Prevention and Control, 2019)
	2016	79.5	1,642							(European Centre for Disease Prevention and Control, 2019)
	2015	64.4	1,328							(European Centre for Disease Prevention and Control, 2019)
	2014	57.4	1,184							(European Centre for Disease Prevention and Control, 2019)
Spain	2020		6,891							(European Food Safety Authority, 2021b)
	2019		9,723							(European Food Safety Authority, 2021a)
	2018		18,411							(European Food Safety Authority, 2021a)
	2017		18,860							(European Centre for Disease Prevention and Control, 2019)
	2016		15,542							(European Centre for Disease Prevention and Control, 2019)
	2015	63.3	13,227							(European Centre for Disease Prevention and Control, 2018a)
	2014	54.9	11,481							(European Centre for Disease Prevention and Control, 2018a)
Sweden	2020	33.3	3,435							(European Food Safety Authority, 2021b)
	2019	65.4	6,693							(National Veterinary Institute, 2020)
	2018	80.4	8,132							(National Veterinary Institute, 2019)
	2017	106.1	10,608							(European Centre for Disease Prevention and Control, 2019)
	2016	111.9	11,021							(European Centre for Disease Prevention and Control, 2019)
	2015	85.9	9,180							(European Centre for Disease Prevention and Control, 2019)
	2014	84.9	8,228							(European Centre for Disease Prevention and Control, 2019)
Switzerland	2020	71.7	6,200							(European Food Safety Authority, 2021b)
	2019	84.2	7,223							(European Food Safety Authority, 2021b)
	2018	90.1	7,675							(European Food Safety Authority, 2021b)
	2017	85.4	7,221							(European Food Safety Authority, 2021b)
	2016	95.4	7,984							(European Food Safety Authority, 2021b)
	2015	84.5	7,070							(European Food Safety Authority, 2019a)
	2014	91.5	7,571							(European Food Safety Authority, 2019a)
United Kingdom	2019	88.1	58,718							(European Food Safety Authority, 2021a)
	2018	98.4	65,246							(European Food Safety Authority, 2021a)
	2017	96.2	63,304							(European Centre for Disease Prevention and Control, 2019)
	2016	90.1	58,911							(European Centre for Disease Prevention and Control, 2019)
	2015	92.2	59,797							(European Centre for Disease Prevention and Control, 2019)
	2014	103.7	66,716							(European Centre for Disease Prevention and Control, 2019)
**Oceania**										
Australia (All jurisdictions except NSW)	2016	146.8	24,164				0-4 (251.1)	1.2:1	Summer	(Bell et al., 2021; NNDSS Annual Report Working Group, 2021)
	2015	139.6	22,573				0–4 (males: 252.4; females: 181.6)	1.2:1		(NNDSS Annual Report Working Group, 2019)
	2014	124.9	19,931				0–4 (males: 241.9; females: 177.2)	1.2:1		(NNDSS Annual Report Working Group, 2016)
Australia (NSW only^&^)	2018	113.5	9,070				<5 (10%; 167.07)	1.2:1	Warmer mons	(Communicable Diseases Branch, 2019)
New Zealand	2019	126.1	6,202				1-4 (242.7)	146.7 vs 106.1	Early summer	(The Institute of Environmental Science and Research Ltd, 2021)
	2018	143.7	6,957				1-4 (302.9)	160.7 vs 124.6	Early summer	(The Institute of Environmental Science and Research Ltd, 2020)
	2017	135.2	6,482				1-4 (257.9)	151.9 vs 119.0	Early summer	(The Institute of Environmental Science and Research Ltd, 2019)
	2016*	158.9	7,456				1-4 (273.6)	177.3 vs 141.0		(The Institute of Environmental Science and Research Ltd, 2017)
	2015	135.3	6,218				1-4 (258.7)	153.6 vs 117.6	Early summer	(The Institute of Environmental Science and Research Ltd, 2016)
	2014	150.3	6,776				1-4 (262.1)	172.8 vs 128.3	Summer	(The Institute of Environmental Science and Research Ltd, 2015)
**North America**										
Canada	2019	27.2	10,237							(Public Health Agency of Canada, 2021)
	2018	27.6	10,236	88%^	7%^	5%^	1 – 4 (35.4)	30.1 vs 25.2	Summer	(Public Health Agency of Canada, 2019; Public Health Agency of Canada, 2021)
	2017	28.5	10,401	90%^	8%^	2%^	20-24 (35.8)	31.1 vs 25.8	Summer	(Public Health Agency of Canada, 2018b; Public Health Agency of Canada, 2021)
	2016	27.5	9,923	91%^	5%^	4%^	1 – 4 (35.5)	30.6 vs 24.4	Summer	(Public Health Agency of Canada, 2018a; Public Health Agency of Canada, 2021)
	2015	25.4	9,079							(Public Health Agency of Canada, 2021)
	2014	28.6	10,121							(Public Health Agency of Canada, 2021)
United States	2020	14.4	7,208							(Ray et al., 2021)
	2019	19.5	9,731							(Tack et al., 2020)
	2018	19.5	9,723							(Tack et al., 2019)
	2017	19.2	9,421							(Marder et al., 2018)
	2016	17.4	8,547							(Marder et al., 2017)
	2015	12.8	6,289	2,437/2,767^	216/2,767^	114/2,767^	<5 (20.8)	14.4 vs 11.3	Summer	(Centers for Disease Control and Prevention, 2017)
	2014	13.3	6,465	2,484/2,825^	259/2,825^	82/2,825^	<5 (22.1)	14.7 vs 11.9	Summer	(Centers for Disease Control and Prevention, 2014)

Age group and season with highest incidence rate. Gender: male to female ratio or incidence rate for male versus female are noted. M: male. F: female. *Disruption of seasonal pattern due to outbreak. ^&^Notifiable in NSW since 2017. ^Only subset of cases has species confirmed. ^#^Case numbers were from outbreaks only. NSW: New South Wales. -Information not available.

#### 3.1.1 Asia

National surveillance data of campylobacteriosis were available in three Asian countries namely Japan, Korea and Singapore.

##### 3.1.1.1 Japan

National data of campylobacteriosis in Japan were available for years 2014 to 2018 from the Food Poisoning Statistics, Ministry of Health, Labour and Welfare of Japan. The data were interpreted in English in related publications (Vetchapitak and Misawa, 2019; Yoshikura, 2020). The national case numbers of foodborne infection reported each year varied from 1,893 to 3,272 (**Table 1**) (Vetchapitak and Misawa, 2019).

Campylobacteriosis in Japan was mainly acquired through outbreaks occurring in restaurants (Yoshikura, 2020). For example, in 2018, there were 278 outbreaks totalling to 1,830 cases of *Campylobacter* infection in Japan, of which 262 (94.2%) outbreaks were due to dining in restaurants (Yoshikura, 2020). Similar infection patterns were also seen in other years. Foodborne outbreaks caused by *Campylobacters* in Japan increased from 212 in year 2014 to 278 in year 2018 (Yoshikura, 2020). About 15-28% of *Campylobacter* infection outbreaks were related to the consumption of chicken meat (Yoshikura, 2020). For example, in 2016, a *Campylobacter* foodborne outbreak happened in Fukuoka city in which 266 individuals suffered from gastroenteritis due to the consumption of sushi topped with undercooked chicken meat (Asakura et al., 2017).

Gastroenteritis caused by *Campylobacter* species in Japan was found to be most prevalent between May and July. Males were found to have a higher incidence rate reported than females, peaking in individuals aged 10 to 20 years (Vetchapitak and Misawa, 2019).

*Campylobacter* was the leading bacterial enteric pathogen causing foodborne diseases in Japan, causing far more infections than other foodborne bacterial pathogens such as *Salmonella* and *Vibrio parahaemolyticus*(Vetchapitak and Misawa, 2019). For example, in 2018, 1,830 cases of foodborne infection were due to *Campylobacter*, while 619 cases were due to *Salmonella* and 218 cases due to *V. parahaemolyticus*(Yoshikura, 2020). A similar pattern was seen in other years.

*C. jejuni* was the main causative species in *Campylobacter* caused gastroenteritis in Japan (Vetchapitak and Misawa, 2019). However, other *Campylobacter* species causing human infections were also reported. A study by Hatanaka et al. examined the presence of *Campylobacter* in 586 stool samples collected from paediatric patients with diarrhea between 2013 and 2015. They found that 48.3% (283/586) of samples were positive for *Campylobacter* genus specific polymerase chain reaction (PCR), among which 51.9% (147/283) were surprisingly positive for *C. ureolytius*, indicating in addition to *C. jejuni*, emerging *Campylobacter* species also contribute to *Campylobacter* induced gastroenteritis in this area (Hatanaka et al., 2017). *C. ureolyticus* was previously detected in patients with inflammatory bowel disease (Zhang et al., 2009).

##### 3.1.1.2 Korea

National data in Korea were available for years 2014-2019 from the Korea epidemiological investigation of infectious diseases annual reports (Korea Disease Control and Prevention Agency, 2015; Korea Disease Control and Prevention Agency, 2016; Korea Disease Control and Prevention Agency, 2017; Korea Disease Control and Prevention Agency, 2018; Korea Disease Control and Prevention Agency, 2019; Korea Disease Control and Prevention Agency, 2021). The annual case numbers of *Campylobacter* infection outbreaks in Korea varied greatly. *Campylobacter* infection cases were the lowest in 2017 (103 cases) and highest in 2016 (902 cases) (**Table 1**).

Outbreaks of food and waterborne diseases caused by *Campylobacter* were found to peak during late spring and summer from May to August which contributed to 40-80% of all outbreaks (**Table 1**) (Korea Disease Control and Prevention Agency, 2015; Korea Disease Control and Prevention Agency, 2016; Korea Disease Control and Prevention Agency, 2017; Korea Disease Control and Prevention Agency, 2018; Korea Disease Control and Prevention Agency, 2019; Korea Disease Control and Prevention Agency, 2021). The common places of outbreaks included workplaces, militaries, schools, and restaurants (Korea Disease Control and Prevention Agency, 2015; Korea Disease Control and Prevention Agency, 2016; Korea Disease Control and Prevention Agency, 2017; Korea Disease Control and Prevention Agency, 2018; Korea Disease Control and Prevention Agency, 2019; Korea Disease Control and Prevention Agency, 2021). For example, in 2019, 12 outbreaks were reported, among which four originated from workplaces, three from school, three from restaurants and two from militaries (Korea Disease Control and Prevention Agency, 2021).

*C. jejuni* was usually the third most common enteric bacterial pathogen in Korea. In 2019, the case numbers of *Campylobacter, Salmonella* and pathogenic *E. coli* infections were 270, 606 and 361, respectively (Korea Disease Control and Prevention Agency, 2021). An exception was the year 2016, where 18 outbreaks caused by *Campylobacter* were noted, which contributed to the highest yearly *Campylobacter* cases (902 cases) and made *Campylobacter* the leading bacterial enteric pathogen in that year (**Table 1**) (Korea Disease Control and Prevention Agency, 2017).

##### 3.1.1.3 Singapore

The latest data from the Communicable Diseases Surveillance in Singapore for foodborne disease was reported in 2018, in which the reported incidence of *Campylobacter* caused gastroenteritis was 7.6 in 100,000 population, similar to that in 2017 and 2016 which were 8.8 and 7.6 in 100,000 population, respectively (**Table 1**) (Ministry of Health Singapore, 2015; Ministry of Health Singapore, 2016; Ministry of Health Singapore, 2017). The yearly cases between 2014-2018 were approximately 430, among which 80-85% were caused by *C. jejuni*, 4-7% by *C. coli* and 10-13% by other unspecified *Campylobacter* species. Children less than four years of age were found to have the highest reported incidence of *Campylobacter* gastroenteritis, being 71.7 in 100,000 population in 2018. There was no obvious gender pattern with the male to female ratio being 1.5:1 in 2018, 1.2:1 in 2017 and 1:1.4 in 2015. Specific information on *Campylobacter* infection outbreaks was not available, but 44.5% (192/431) of foodborne infection notifications were classified as outbreaks in 2016, 93.9% (214/228) in 2015, and 94.4% (284/301) in 2014, indicating outbreaks are the main form of foodborne diseases in Singapore (Ministry of Health Singapore, 2015; Ministry of Health Singapore, 2016; Ministry of Health Singapore, 2017).

*Campylobacter* was the second most common foodborne bacterial pathogen in Singapore following *Salmonella*. In 2018, the reported incidence of salmonellosis was 28.7 per 100,000 population. The reported incidence of foodborne diseases such as typhoid and paratyphoid fever were 0.8 and 0.3, respectively. There were only two imported cases of cholera; therefore the reported incidence of cholera was zero in 2018 (Ministry of Health Singapore, 2017).

A recent study examining the aetiology of acute gastroenteritis using stool specimens collected between February and October 2016 reported that *Campylobacter*(9.4%, 28/299) was the second leading bacterial pathogen detected, following *Salmonella*(19.1%, 57/299) (Koo et al., 2022). Chau et al. examined the presence of enteric pathogens in faecal samples collected from adult patients with acute diarrhea between October 2013 and January 2014 using multiplex PCR (Chau et al., 2015). They reported that the most frequently detected microorganisms were norovirus genogroup II (11%, 11/100), *Aeromonas*(9%, 9/100) and *Campylobacter*(5%, 5/100) (Chau et al., 2015).

#### 3.1.2 Europe

Campylobacteriosis in Europe is monitored by the European Centre for Disease Prevention and Control. According to the European Union One Health 2020 Zoonoses Report, campylobacteriosis was the leading cause of human zoonoses, consisting of 120,946 cases, representing more than 60% of all reported zoonotic disease cases (European Food Safety Authority, 2021a). Other major zoonoses included salmonellosis (52,702 cases), yersiniosis (5,668) and Shiga toxin-producing *E. coli*(STEC) infections (4,446 cases) (European Food Safety Authority, 2021b). Subsets of the confirmed cases of campylobacteriosis had available information on *Campylobacter* species. In 2020, 64.7% of confirmed cases had available information on *Campylobacter* species, among which 88.1% were caused by *C. jejuni*, 10.6% caused by *C. coli*, and the remaining caused by *C. fetus*(0.16%), *C. upsaliensis*(0.11%), *C. lari*(0.09%), and other *Campylobacter* species (0.94%) (**Table 1**) (European Food Safety Authority, 2021b). This *Campylobacter* species distribution pattern was consistent with previous years (European Food Safety Authority, 2019a; European Food Safety Authority, 2021a).

In Europe, children under five years of age contributed to approximately 13% of all confirmed cases. There was a higher rate in males than females (1.2:1) (European Centre for Disease Prevention and Control, 2018b; European Centre for Disease Prevention and Control, 2018a; European Centre for Disease Prevention and Control, 2019). A clear seasonal distribution of campylobacteriosis in Europe was seen with most cases being reported during summer months (**Table 1**) (European Centre for Disease Prevention and Control, 2018b; European Centre for Disease Prevention and Control, 2018a; European Centre for Disease Prevention and Control, 2019).

In addition to the summer peak, a smaller but distinct winter peak was also observed in European countries including Austria, Belgium, Finland, Germany, Luxembourg, the Netherlands, Switzerland and Sweden (European Food Safety Authority, 2021a). Risk factors contributing to the winter peak included consumption of meat fondue or table-top grilling during the festive season as well as increased travel during Christmas and New Year (Bless et al., 2017). However this winter peak was not observed in Denmark, France, Ireland, Italy, Norway and the United Kingdom (Bless et al., 2017).

Sporadic cases were the main form of campylobacteriosis in Europe. In 2020, among the 120,946 cases reported, 1,319 cases were caused by 317 foodborne outbreaks, contributing to only 1% of total number of cases. Similar proportions were also observed in previous years. Among ready-to-eat food, main categories with occurrence of *Campylobacter* included fruit, vegetables and juices (36.6%), milk and milk products (24.2%), meat and meat products (12.9%) and salads (10.2%). Among non-ready-to-eat food, meat and meat products (79.7%) were the main contributor, followed by milk and milk products (5.4%), and fruit, vegetables and juices (3.3%).

##### 3.1.2.1 Austria

The reported incidence of campylobacteriosis in Austria was 76.6 per 100,000 in year 2014, followed by an increasing trend from 2015 to 2018 with the reported incidence being 72.9 to 90.7 per 100,000. In 2019, the reported incidence was 74.2 per 100,000, which further decreased to 60.7 per 100,000 in 2020 (**Table 1**). *Campylobacter* was the leading bacterial enteric pathogen in Austria. The reported incidence of other bacterial pathogens in 2020 including *Salmonella*, STEC, *Yersinia*, and *Listeria monocytogenes* were 9.2, 3.2, 1.4 and 0.46 per 100,000, respectively (European Food Safety Authority, 2021b).

##### 3.1.2.2 Belgium

The highest reported incidence of campylobacteriosis in Belgium was in year 2016, being 88.9 per 100,000 population (**Table 1**). The reported incidence then gradually decreased and reached 48.6 per 100,000 in 2020 (European Food Safety Authority, 2021b). *Campylobacter* was the leading bacterial enteric pathogen in Belgium. In 2020, the reported incidence of other bacterial pathogens causing gastroenteritis including *Salmonella, Yersinia*, STEC, and *L. monocytogenes* were 13.8, 2.3, 0.73, and 0.59 per 100,000, respectively.

##### 3.1.2.3 Bulgaria

In Bulgaria, the reported incidence of *Campylobacter* infection during 2014 and 2020 varied between 1.8 and 3.3 per 100,000 population, and the number of annual cases ranged between 144 and 229 (**Table 1**) (European Centre for Disease Prevention and Control, 2019; European Food Safety Authority, 2021a). *Campylobacter* is not the leading zoonotic pathogen in Bulgaria. In 2020, *Salmonella* was the leading bacterial pathogen with the reported incidence being 2.7 per 100,000 population, followed by *Campylobacter*(1.8), *Trichinella*(0.19), *L. monocytogenes* and *Yersinia*(0.06). A recent study examining the bacterial etiological agents causing acute diarrhea in Bulgaria between 2014 and 2018 also showed that *Salmonella* was the leading bacterial pathogen, causing 44% of cases examined, followed by *Shigella*(26%), STEC (20%), and *Campylobacter*(8%) (Pavlova et al., 2019).

Although the reported incidence of campylobacteriosis remained low in Bulgaria, a research study performed between February 2014 and January 2015 examining patients with diarrheal syndrome reported high detection rate of *Campylobacter* from their stool samples, in which 43% (40/93) of the samples examined were PCR positive for *Campylobacter*(Pavlova et al., 2016). Furthermore, a recent study examining the presence of *Campylobacter* in children with acute diarrhea under five years of age reported a high infection rate, raising concerns regarding *Campylobacter* infection in paediatric patients. *Campylobacter* was found in 37.8% (139/368) of faecal samples examined, among which 33.2% were *C. jejuni* and 4.6% were *C. coli*(Pavlova et al., 2020). Boys were found to have a significant higher positive rate than girls (64 versus 36%, respectively). Furthermore, children between three and five years of age were found to have a significantly higher infection rate than other age groups, accounting for half of the positive cases. Authors suggested that this might be due to a more varied diet in older children (Pavlova et al., 2020).

##### 3.1.2.4 Croatia

The reported incidence of *Campylobacter* infection remained relative steady in Croatia, ranging from 38.8 per 100, 000 in 2016 to 42.2 in 2019. In 2020, the reported incidence dropped to 26 per 100,000, however still remained the leading bacterial enteric pathogen (**Table 1**) (European Food Safety Authority, 2021b). The reported incidence of other enteric agents including *Salmonella*, *Yersinia*, STEC, and *L. monocytogenes* in the year 2020 were 19.4, 0.27, 0.20 and 0.12, respectively (European Food Safety Authority, 2021b).

##### 3.1.2.5 Cyprus

Cyprus has a low reported incidence of campylobacteriosis, ranging from 4.7 per 100,000 in 2014 to 2.4 in 2019, further dropping to 2.0 in 2020 (**Table 1**). *Salmonella* was the leading bacterial enteric pathogen in Cyprus with a reported incidence of 7.9 in 2020, followed by *Campylobacter*(2.0) and *L. monocytogenes*(0.23). No cases of STEC and *Yersinia* infection were reported in 2020.

##### 3.1.2.6 Czech Republic

Czech Republic has the highest reported incidence of campylobacteriosis among all European countries. The reported incidence increased from 197.4 per 100,000 in 2014 to 230.0 in 2017, then dropped to 215.0 in 2019 before further decreasing to 163.8 in 2020 (**Table 1**) (European Food Safety Authority, 2021b). *Campylobacter* was the leading bacterial enteric pathogen in Czech Republic. Additionally, its reported incidence of *Salmonella* infection was also the highest in Europe, being 98.4 in 2020. The reported incidence rates of *L. monocytogenes*, *Yersinia* and STEC infections in Czech Republic were 0.15, 4.1 and 0.3, respectively in 2020 (European Food Safety Authority, 2021b).

##### 3.1.2.7 Denmark

There has been a continuous increase in *Campylobacter* infection in Denmark since 2012 (Statens Serum Institut, 2019). The reported incidence of campylobacteriosis in 2014 was 67.0 per 100,000 population, which increased to 82 in 2016, and to 93 in 2019 (**Table 1**). A rapid decrease in *Campylobacter* infection was noted in 2020, with a reported incidence of 64.3 and the case numbers of 3,742 (National Food Institute, 2021).

The majority of the *Campylobacter* infections in Denmark were sporadic, in which only 5% of the cases in 2020 were due to outbreaks. In May 2020, a large outbreak consisting of 161 cases was reported on Bornholm, most likely due to consumption of a particular brand of pasteurised milk. Another two smaller outbreaks involving 20 and 18 cases due to the consumption of Danish-produced chicken and unknown causes were also reported (National Food Institute, 2021). In 2019, a total of 5,389 cases were notified, which was the highest number ever recorded (Statens Serum Institut, 2019; Joensen et al., 2021). Among those cases, 701 human clinical isolates were analysed, of which 93% (652/701) were *C. jejuni* and 7% were *C. coli*(Joensen et al., 2021). Furthermore, through whole genome sequencing and cluster analysis, it was found that one third of all clinical isolates (31%, 219/701) matched isolates from chicken meat, suggesting chicken could be a major source of foodborne outbreaks (Joensen et al., 2021).

There has been a consistent gender predominance of *Campylobacter* infection in Denmark, where males were more often infected than females. Elders more than 85 years of age and young adults between 20 and 29 were shown to have a high reported incidence among all age groups. Between 2016 and 2019, the highest reported incidence was found in Bornholm where more than 200 cases per 100,000 inhabitants were reported (Statens Serum Institut, 2018; Statens Serum Institut, 2019).

A prospective study conducted in 2016 among Danish subjects aged 1-30 years old identified several determinants of sporadic *Campylobacter* infections. Such risk factors included bathing in fresh water, contact with beach sand, owning a pet dog with diarrhea, eating minced beef or chicken, as well as foreign travel to Asia, Africa or Turkey and eating street food (Kuhn et al., 2018).

*Campylobacter* was the leading bacterial enteric pathogen in Denmark, followed by *Salmonella*, STEC, *Yersinia* and *L. monocytogenes*. The reported incidence rates of *Salmonella*, STEC, *Yersinia* and *L. monocytogenes* infections in 2020 were 10.5, 7.6, 7.1 and 0.76, respectively (European Food Safety Authority, 2021b).

##### 3.1.2.8 Estonia

The reported incidence of campylobacteriosis in Estonia remained steady during the past years, ranging from 21.7 to 31.2 per 100,000 population during 2014 to 2019. In 2020, the reported incidence dropped slightly to 19.9 (**Table 1**) (European Food Safety Authority, 2021b). *Campylobacter* was the leading bacterial enteric pathogen in Estonia, followed by *Salmonella*(6.8), *L. monocytogenes*(3.3), STEC (0.75), and *Yersinia*(0.23) (European Food Safety Authority, 2021b).

##### 3.1.2.9 Finland

Finland had a decreasing trend of campylobacteriosis during the period between 2014 and 2019, as the reported incidence decreased from 89.7 per 100,000 population in 2014 to 79.4 in 2019, then rapidly dropped to 37.5 in 2020 (**Table 1**) (European Food Safety Authority, 2021b). *Campylobacter* outbreaks were rarely reported in Finland. In 2019, two outbreaks consisting of six cases were reported, broiler meat and mixed food were found to be the source of contamination; whereas in 2018, three outbreaks were reported consisting of a total number of 19 cases; mixed food were found to be the vehicle of transmission (European Food Safety Authority, 2019b; European Food Safety Authority, 2021c).

In 2019, Finland reported the highest proportion of travel associated cases (49.2%) among all European countries, whereas in other European countries, the proportion of travel associated cases accounted to less than 26% (European Food Safety Authority, 2021b).

*Campylobacter* was the leading bacterial enteric pathogen in Finland, followed by *Salmonella*(reported incidence of 9.3 per 100,000 in 2020), *Yersinia*(7.0), STEC (3.2), and *L. monocytogenes*(1.7) (European Food Safety Authority, 2021b).

##### 3.1.2.10 France

A continuously increasing trend of campylobacteriosis was observed in France from 2014 to 2020, with the reported incidence increasing from 45.2 per 100,000 in 2014 to 57.5 in 2019 and 58.8 in 2020 (**Table 1**) (European Food Safety Authority, 2021b). Along with Luxembourg, France was one of the two European countries that observed an increase in the number of cases in 2020 as compared to 2019 (European Food Safety Authority, 2021b). *Campylobacter* was the leading bacterial enteric pathogen in France, followed by STEC (reported incidence of 21.9 in 2020), *Salmonella*(9.3), and *L. monocytogenes*(0.5) (European Food Safety Authority, 2021b).

##### 3.1.2.11 Germany

There has been a decreasing trend of the reported incidence of campylobacteriosis in Germany from 2014 to 2020, from 87.4 per 100,000 population in 2014 to 73.8 in 2019, which further dropped to 55.8 in 2020 (**Table 1**) (European Centre for Disease Prevention and Control, 2019; European Food Safety Authority, 2021a). *Campylobacter* was the leading bacterial enteric pathogen in Germany, in which its reported incidence was far higher than other pathogens such as *Salmonella*(reported incidence of 10.4 in 2020), *Yersinia*(2.2), STEC (1.7) and *L. monocytogenes*(0.65) (European Food Safety Authority, 2021b).

##### 3.1.2.12 Greece

Greece is one of the countries with a low reported incidence of campylobacteriosis in Europe. The reported incidence rates in 2018, 2019 and 2020 were 3.3. 3.4 and 2.0 per 100,000 population, respectively, with case numbers being 357, 366 and 218, respectively (**Table 1**) (European Food Safety Authority, 2021b; European Food Safety Authority, 2021a). *Salmonella* infection in Greece was more common than that of *Campylobacter*, with the reported incidence being 3.6 in 2020, and 6.0 in 2018 and 2017. The reported incidence of infections caused by other enteric bacterial pathogens such as *L. monocytogenes* and STEC remained low, which were 0.2 and 0.05 in past years, respectively (European Food Safety Authority, 2021b).

A large waterborne outbreak was notified in Northern Greece in 2019 in which a total of 638 cases of gastroenteritis were recorded (Tzani et al., 2020). Eleven of these cases were examined for the presence of enteric pathogens and eight of them (8/11) were PCR positive for *C. jejuni*. Additionally, other pathogens including norovirus, *E. coli* O15 and EPEC were also identified.

##### 3.1.2.13 Hungary

There has been a decrease in the reported incidence of campylobacteriosis in Hungary during 2014-2019, from 85.5 per 100,000 population in 2014 to 65.5 in 2019, which further dropped to 45.7 in 2020 (**Table 1**) (European Food Safety Authority, 2021b). *Salmonella* was the second common enteric pathogen causing zoonotic gastroenteritis, with the reported incidence being 30.3, consisting of 2,964 cases in 2020. The reported incidence rates of other bacterial enteric pathogens including *L. monocytogenes*, *Yersinia*, and STEC were much lower, with the reported incidence in 2020 being 0.33, 0.26 and 0.08, respectively (European Food Safety Authority, 2021b).

##### 3.1.2.14 Iceland

The rate of *Campylobacter* infection in Iceland has slightly decreased since 2014, with the reported incidence being 43.6 per 100,000 in 2014, 38.1 in 2019, further decreasing to 26.1 in 2020. The case numbers were 142 in 2014, 136 in 2019 and 95 in 2020, respectively (**Table 1**) (European Food Safety Authority, 2021b). *Campylobacter* was the leading bacterial enteric pathogen in Iceland. The reported incidence rates of gastroenteritis caused by other zoonotic enteric bacterial pathogens including *Salmonella*, *L. monocytogenes*, STEC and *Yersinia* were much lower, being 8.8, 1.1, 1.1 and 0.26 per 100,000 population in 2020, respectively (European Food Safety Authority, 2021b).

##### 3.1.2.15 Ireland

The rate of campylobacteriosis remained stable in Ireland for the course between 2014 and 2017, with the reported incidence varying between 52.8 and 58.5 per 100,000 population, with case numbers between 2,451 and 2,786 (**Table 1**) (European Food Safety Authority, 2021b). The reported incidence increased to 63.6 per 100,000 population in 2018 (case number 3,030), but reduced to 56.6 in 2019 (case number 2,777) and further dropped to 48.7 in 2020 (case number 2,419) (European Food Safety Authority, 2021b). During 2015 to 2019 where species information was available for subsets of *Campylobacter* isolates, 86-93% were *C. jejuni*, 6-12% were *C. coli*, whilst the remaining were other *Campylobacter* species (Health Protection Surveillance Centre, 2018; Health Protection Surveillance Centre, 2019; Brehony et al., 2021; Health Protection Surveillance Centre, 2021).

Most of the *Campylobacter* infections in Ireland were sporadic, with only six outbreaks notified in 2017 and five outbreaks in 2018, consisting of 28 and 19 cases, respectively (Health Protection Surveillance Centre, 2018; Health Protection Surveillance Centre, 2019). Known routes of transmission contributing to such outbreaks included foodborne, waterborne, person-to-person or animal contact (Health Protection Surveillance Centre, 2018; Health Protection Surveillance Centre, 2019). In Ireland, the age group of 0-4 years was consistently found to have the highest reported incidence rate (Health Protection Surveillance Centre, 2018; Health Protection Surveillance Centre, 2019; Health Protection Surveillance Centre, 2021). There was also a well-documented seasonal distribution with an early summer peak observed every year.

Campylobacteriosis was the most common form of bacterial gastroenteritis in Ireland (Health Protection Surveillance Centre, 2019). The reported incidence rates of other bacterial pathogens including STEC, *Salmonella*, and *L. monocytogenes* were much lower, being 14.8, 4.3 and 0.12 in 2020, respectively (European Food Safety Authority, 2021b).

##### 3.1.2.16 Italy

According to the European Union One Health 2020 Zoonoses Report, the information of reported incidence was not available due to the lack of information on population coverage. The number of notified *Campylobacter* cases in Italy between 2014 and 2020 varied between 1,060 and 1,633 (**Table 1**) (European Centre for Disease Prevention and Control, 2019; European Food Safety Authority, 2021b; European Food Safety Authority, 2021a).

A recent study by Stanyevic et al. examined the evolving epidemiology on acute gastroenteritis in hospitalised children in Italy. Among the 74 stool samples from 2019 subjected for examination of aetiological agents, 8.1% (6/74) was positive for *C. jejuni*, similar to that from 2012 (12.5%, 8/64) (Stanyevic et al., 2021). Other less prevalent enteric bacterial pathogens reported in 2019 included non-Typhi *Salmonella*(4/74), *Yersinia enterocolitica*(1/74) and *E. coli*(1/74). Adenovirus (9/74), rotavirus (4/74), and norovirus (4/74) were the leading enteric viral pathogens causing paediatric gastroenteritis (Stanyevic et al., 2021). A similar detection rate of *Campylobacter* was reported by another study performed between 2018 and 2020 in which among stool samples from 2,066 children with severe acute gastroenteritis, 9.21% were positive for *Campylobacter*, whereas enteropathogenic *E. coli*(EPEC) (19.14%), *Clostridioides difficile*(14.42%), norovirus (10.36%) and enterovirus (9.44%) were detected more frequently (De Conto et al., 2022). In 2018, Sorgentone et al. reported a large foodborne outbreak in kindergarteners and primary schools in Pescara, Italy (Sorgentone et al., 2021). In this study, a total of 224 human stools were collected; 40.6% (91/224) were positive for *Campylobacter* using real-time polymerase chain reaction (rt-PCR), and 62 C*. jejuni* strains were isolated from 60 patients. An investigation of possible causes suggested a failure in the pasteurisation process of the milk used for cheese.

##### 3.1.2.17 Latvia

Although Latvia was one of the European countries with a low reported incidence of *Campylobacter* infection, there has been an increasing trend over the period between 2014 and 2020 (**Table 1**). The reported incidence in 2014 and 2015 was 1.8 and 3.7 per 100,000 population, respectively, whereas in 2019 and 2020 was 6.9 and 5.5, respectively. The case numbers increased from 37 in 2014 to 133 in 2019 and 104 in 2020 (European Centre for Disease Prevention and Control, 2019; European Food Safety Authority, 2021b; European Food Safety Authority, 2021a). A study examining the presence of *Campylobacter* species in sporadic gastroenteritis cases during 2015 and 2016 reported low isolation rates of *Campylobacter* species, in which *C. jejuni* and *C. coli* were isolated from 5% (23/434) and 0.2% (1/434) of human stool samples, respectively (**Table 2**) (Meistere et al., 2019).

**Table 2:** *Campylobacter* infection in countries without national surveillance program.

Country	Year	Region	*Campylobacter*	*C. jejuni*	*C. coli*	Other *Campylobacter* species	Peak age(range)	Gender	Season	Method	Reference
**Africa**											
Egypt	2019	Beni-Suef		48% (25)						Isolation & PCR	(Zeinhom et al., 2021)
	2018	Cairo, Giza, Fayoum and Qalyubia	38.09% (105)	35.2% (105)	2.9% (105)					PCR	(Barakat et al., 2020)
	2017-2018	Sharkia		30% (100)						Isolation & PCR	(Ammar et al., 2020)
	2017-2018	Ismailia		31.2% (80)							(Abdelmageed et al., 2021)
	2015-2018	Zagazig		4.1% (270)						Isolation & PCR	(Abd El-Hamid et al., 2019)
	2015-2016	Cairo, Giza, Fayoum, Qalubia & Minya		17.33% (75)						PCR	(Ghoneim et al., 2020)
	2013-2014	Fayuom, Cairo, Qaluobia, Bin-suef and Assuit		21.5% (93)						Isolation & PCR	(Elgabry et al., 2016)
	2013-2014	Mansoura	7.84% (102)				(18-50y)			PCR	(Ramadan et al., 2015)
	2013-2014	Zagazig		4.1% (246)						Isolation & rtPCR	(Ahmed et al., 2015)
Ethiopia	2019	Arba Minch	4.4% (180)				(≥15y)			Isolation	(Ayele et al., 2020)#
Ghana	2015-2016	Accra	50.7% (140)		50.7% (140)		31-50y (20-80y)	Female		Isolation & PCR	(Forson et al., 2020)#
Madagascar	2011-2014	Moramanga and Antananarivo	1% (199)				(<5y)			Isolation	(Randremanana et al., 2016)^
Malawi	2012-2015	Blantyre	16.5% (684)				(<5y)			rtPCR	(Iturriza-Gómara et al., 2019)^
South Sudan	2017	United Kingdom military personnel in South Sudan	0.8% (127)				(18->45y)			PCR	(Biswas et al., 2019)^&^
**Asia**											
Bangladesh	2019-2020	Mymensingh	31.5% (330)	21.8% (330)	9.6% (330)		0-5y (0->60y)	Female		Isolation & PCR	(Rahman et al., 2021)
	2019-2020	Dhaka	3.6% (2135)				(≥5y)			Isolation	(Garbern et al., 2021)
China	2019	Beijing	85.7% (12/14)							rtPCR	(Li et al., 2020b)*
	2017-2019	Wenzhou	10.5% (850)	9.3% (850)	1.2% (850)		(6m-91y)			Isolation	(Zhang et al., 2020a)
	2009-2018	31 provinces		0.87% (90391)	0.15% (89049)		1-17y (<5->60y)	Female	Spring	Isolation	(Wang et al., 2021)
	2017-2018	Beijing	7.8% (2945)				6–17y (<5->65y)	Male	Autumn	Isolation	(Zhang et al., 2020b)
	2018	Beijing		90.0% (11)			(15-40y)			rtPCR	(Qu et al., 2019)*
	2018	Hangzhou		55.6% (27)						Isolation	(Yu et al., 2020)*
	2016-2017	Beijing	7% (370)	6.5% (370)	0.5% (370)		76–87y (15-87y)	Male	Winter	Isolation	(Li et al., 2018)
	2012-2016	Shanghai	1.2% (8797)	1.1% (8797)	0.08% (8797)		(18->67)			Isolation	(Gong et al., 2018)
	2014-2015	Shenzhen		4.9% (412)			5-9y (1m-78y)			Isolation	(Shen et al., 2016)
	2014-2015	Wuhan		2.9% (381)			2-5y (<5y)			Isolation	(Zhu et al., 2016)^
	2011-2014	Henan	0.13% (755)				(<5y)			Isolation	(Wang et al., 2015)^
	2010-2014	Southeast	0.0003% (3175)				(<5->65y)			Isolation	(Chen et al., 2019)
	2009-2014	Mainland	0.5% (5967)				(>65y)			Isolation	(Zhang et al., 2017)
	2009-2014	Zhejiang	0.4% (2318)				<1y (<5y)		Summer	Isolation	(Zheng et al., 2016)^
India	2019-2020	Vellore	12% (400)	4.3% (400)	2.5% (400)		31-40y (0-80y)			Isolation & rtPCR	(Lakshmi Ss et al., 2022)
	2016-2017	Odisha	16.77% (310)				2-5y (0->5y)	Female		PCR	(Mohakud et al., 2019)^
	2014-2016	Northeast	10.1% (407)	8.1% (407)			<2y (<5y)	Male	Summer	PCR	(Borkakoty et al., 2020)^
Iran	2017	Mazandaran		27% (74)						Isolation & PCR	(Divsalar et al., 2019)
	2016	East Azerbaijan	35.4% (223)	27.8% (223)	10.8% (223)		18-30y (18-70y)	Male		Isolation & PCR	(Ranjbar et al., 2017)
	2015	Central Iran		33% (230)			1-3y (<1-10y)	Male		PCR	(Abbasi et al., 2019)^
	2014-2015	Semnan	8.6% (419)				6-12y (<2->6y)	Female		Isolation	(Mazaheri et al., 2016)^
	2013-2014	Hamadan	10% (120)				(<10y)			Isolation & PCR	(Rastyani et al., 2015)^
	2012-2014	Tehran	3.6% (980)	3.4% (980)	0.2% (980)				Summer	Isolation & PCR	(Shams et al., 2017)^
Iraq	2017	Thi-Qar	10.9% (155)				(<5y)			PCR	(Harb et al., 2019)^
Lebanon	2018	South	12% (291)				>5y (1m-12y)	Male		Isolation	(Ghssein et al., 2021)^
	2016-2017	8 districts	21.5% (1000)	17.9% (1000)	3% (1000)		<12 (<12->65)	Male	Summer	Isolation & PCR	(Ibrahim et al., 2019)
Nepal	2017-2018	Kathmandu	56.8% (303)				<6m (<5y)			Isolation	(Bhattarai et al., 2020)^
	2011-2014	International travelers to Nepal		16% (480)	2.7% (480)	*C. concisus* 31.3% (83); *C. ureolyticus* 7.2% (83)				PCR	(Serichantalergs et al., 2017)^&^
	2012-2014	International travelers to Nepal		20% (433)						Isolation & rtPCR	(Murphy et al., 2019)^&^
Pakistan	2014-2015	Rawalpindi, Islamabad, Lahore, Peshawar, Khairpur, Mardan and Nowshera		54.6% (150)						Isolation & PCR	(Noreen et al., 2020)^
	2014	Rawalpindi & Islamabad	52% (500)	48.2% (500)			6-11m	Male	Summer	rtPCR	(Sadiq et al., 2019a)^
Thailand	2016-2018	Nationwide	10.8% (370)				<5y			Isolation & rtPCR	(Okada et al., 2020)
	2013-2017	United States military personnel in Thailand	43.8% (248)	25% (48)	18.8% (48)					Isolation, rtPCR & ELISA	(Lurchachaiwong et al., 2020)^&^
	2012-2014	International travelers to Thailand	31.2% (154)							rtPCR	(Lertsethtakarn et al., 2018)^&^
	2011-2014	International travelers to Thailand		24.9% (173)	5.2% (173)	*C. concisus* 11.5% (26); *C. ureolyticus* 7.7% (26)				PCR	(Serichantalergs et al., 2017)^&^
United Arab Emirates	2017-2019	Al Ain	1.9% (203)				(<5y)			rtPCR	(Alsuwaidi et al., 2021)^
**Oceania**											
Papua New Guinea	2013-2014	Hela, Eastern Highlands, Madang and Central Provinces	33.1% (118)				(1m-69y)			rtPCR	(Abdad et al., 2020)
**Europe**											
North Macedonia	2016-2017	Skopje	2.5% (3820)	2.2% (3820)	0.3% (3820)		<15y (<15->50y)			Isolation	(Trajkovska-Dokic et al., 2019)
**South America**											
Colombia	2013-2014	Bucaramanga	3.5% (431)				(<5y)			Isolation	(Farfán-García et al., 2020)^

Gender and season with highest isolation or detection rate were noted. Age group with highest isolation or detection rate (range of ages included in the study). M: month. Y: year. Incidence: expressed as percentage (total number of cases). *Outbreak. ^Pediatric studies. ^&^Traveler’s diarrhea. ^#^HIV patients with diarrhea. If multiple methods were used for detection, the higher detection rate is noted. Studies of sample size less than 10 were excluded. EIA: enzyme immunochromatographic assay. PCR: polymerase chain reaction. rtPCR: real-time polymerase chain reaction.

*Campylobacter* was the second leading bacterial enteric pathogen in Latvia, following *Salmonella*(European Food Safety Authority, 2021b). The reported incidence of salmonellosis in 2020 was 15.5, consisting of 296 cases. Other less prevalent enteric bacterial pathogens such as *Yersinia* and STEC had reported incidence rates of 4.6 and 0.1, respectively in 2020 (European Food Safety Authority, 2021b).

##### 3.1.2.18 Lithuania

The rate of campylobacteriosis in Lithuania remained mostly stable during 2014 to 2020, with the reported incidence being 40.2 per 100,000 individuals in 2014 and 42.3 in 2020 (**Table 1**). A decrease in reported incidence was observed in years between 2017 and 2018, with the reported incidence being 34.8 and 32.7, respectively (European Centre for Disease Prevention and Control, 2019; European Food Safety Authority, 2021b; European Food Safety Authority, 2021a). Infections caused by other enteric pathogens such as *Salmonella* and *Yersinia* had a much lower reported incidence as compared to *Campylobacter*, namely 17.8 and 4.9, respectively in 2020 (European Food Safety Authority, 2021b).

##### 3.1.2.19 Luxembourg

Between 2014 and 2020, the reported incidence of campylobacteriosis in Luxembourg greatly fluctuated from 45.1 per 100,000 individuals in 2015, to more than double (103.8) in 2017 and 2018 (**Table 1**) (European Centre for Disease Prevention and Control, 2019). There was a sharp decrease in the reported incidence (44.1) in 2019 which was due to a surveillance artefact caused by a change in diagnostic methods in private laboratories, resulting in reduced numbers of isolates sent to the national reference laboratory (European Food Safety Authority, 2021a). An electronic laboratory notification system was established in March 2020, and the reported incidence rate in 2020 (116.4) increased back to the level that was comparable to that in 2018 and 2017 (European Food Safety Authority, 2021b).

*Campylobacter* was the leading enteric pathogen in Luxembourg. The reported incidence rates of enteric infections caused by *Salmonella, Yersinia*, and *L. monocytogenes* were much lower, being 14.9, 4.2 and 0.64, respectively, in 2020 (European Food Safety Authority, 2021b).

##### 3.1.2.20 Malta

The trend in *Campylobacter* infection showed variations in Malta between 2014 and 2020, with the incidence varied between 40.4 and 70.0 per 100,000 individuals, and case numbers varied between 206 and 333 (European Centre for Disease Prevention and Control, 2019; European Food Safety Authority, 2021b; European Food Safety Authority, 2021a). *Campylobacter* was the leading bacterial pathogen causing gastroenteritis in Malta, followed by *Salmonella* and *L. monocytogenes*, with the reported incidence rates being 34.2 and 0.97 in 2020, respectively (European Food Safety Authority, 2021b).

##### 3.1.2.21 Netherlands

The reported incidence of campylobacteriosis in Netherlands was on a continuous decreasing trend, from 47.5 per 100,000 population in 2014, to 38.3 in 2016, further dropping to 34.1 in 2019 and 25.2 in 2020 (**Table 1**) (European Centre for Disease Prevention and Control, 2019; European Food Safety Authority, 2021b; European Food Safety Authority, 2021a). Other less prevalent bacterial pathogens including *Salmonella*, STEC, and *L. monocytogenes* had reported incidence rates of 6.2, 1.9 and 0.52 in 2020, respectively (European Food Safety Authority, 2021b).

##### 3.1.2.22 Norway

There has been an increase in *Campylobacter* infections in Norway in the past years, from 44.9 per 100,000 population in 2015 to 73.9 in 2017 and 78.0 in 2019 (**Table 1**). In 2019, Norway recorded a total of 4,154 cases, which was the highest level in the past decade (European Centre for Disease Prevention and Control, 2019; European Food Safety Authority, 2021a). There was a sharp drop in reported *Campylobacter* case numbers (2,422 cases) and incidence (45.1) in 2020 (European Food Safety Authority, 2021b).

A large outbreak of gastroenteritis was notified in Askøy on 6^th^ June 2019 (Hyllestad et al., 2020). *C. jejuni* isolates from 24 clinical samples and four water samples showed identical core genome multilocus sequence typing profiles, indicating the water supply as the source of the outbreak. Another study examining hospitalised individuals due to the same water outbreak showed that among 59 patients who had fecal *Campylobacter* tests taken, all tested positive for *C. jejuni*, either by bacterial cultivation or PCR (Mortensen et al., 2021). Contamination through cracks in the water reservoir most likely had occurred during heavy rainfall, indicating the importance of water safety planning and risk assessment (Hyllestad et al., 2020). A recent publication suggested the use of coagulation and UV radiation as treatments to improve water quality in areas where surface water is used as a source for drinking water production (Herrador et al., 2021).

*Campylobacter* was the leading bacterial enteric pathogen causing gastroenteritis in Norway, followed by *Salmonella*, STEC, *Yersinia* and *L. monocytogenes*, with the reported incidence being 8.2, 6.2, 1.5 and 0.69, respectively in 2020 (European Food Safety Authority, 2021b).

##### 3.1.2.23 Poland

The reported incidence of campylobacteriosis in Poland was low, varying between 1.1 and 2.3 per 100,000 population, in the years between 2014 and 2020 (**Table 1**) (European Centre for Disease Prevention and Control, 2019; European Food Safety Authority, 2021b; European Food Safety Authority, 2021a). Despite the low reported incidence, Poland had a high hospitalisation rate with 76.6% of all reported cases in 2020 were hospitalised (European Food Safety Authority, 2021b). *C. jejuni* was the leading *Campylobacter* species causing campylobacteriosis in Poland. In 2015 and 2016 where species information was available for subsets of cases, *C. jejuni* accounted for 96% and 93% of cases examined, while *C. coli* accounted for 4% and 7%, respectively (Radziszewski et al., 2018). Males were more often infected than females. The age group of 0-4 years was consistently found to be most frequently infected, accounting for more than 70% of all reported cases.

Salmonellosis was more common than campylobacteriosis in Poland which had a reported incidence of 13.7 per 100,000 population in 2020. Other less common enteric bacterial pathogens such as *Yersinia* and STEC had a reported incidence of 0.23 and 0.01, respectively in 2020 (European Food Safety Authority, 2021b).

##### 3.1.2.24 Portugal

Since 2015, the reported incidence of campylobacteriosis in Portugal showed an increasing trend, from 2.6 in 2015 to 5.8 per 100,000 population in 2017, and further increased to 8.6 in 2019 (**Table 1**). However, in 2020, the reported incidence dropped slightly to 7.7. Much lower reported incidence rates of diarrheal disease caused by other bacterial pathogens including *Salmonella*, *L. monocytogenes*, *Yersinia* and STEC were reported in 2020, being 2.5, 0.46, 0.24 and 0.05 per 100, 000 population, respectively (European Food Safety Authority, 2021b).

##### 3.1.2.25 Romania

The reported incidence of campylobacteriosis was low in Romania but showed an increasing trend, from 1.3 per 100,000 population in 2014, to 2.4 in 2017, and further increased to 4.1 in 2019 (**Table 1**) (European Centre for Disease Prevention and Control, 2019; European Food Safety Authority, 2021b; European Food Safety Authority, 2021a). There was a drop of *Campylobacter* infection in 2020 in which the reported incidence was 1.6 (European Food Safety Authority, 2021b).

*Salmonella* was the leading bacterial enteric pathogen causing gastroenteritis in Romania nationally, with the reported incidence varying between 5.9 and 7.6 in years between 2014 and 2019. In 2020, the reported incidence of salmonellosis dropped to 2.1. The reported incidence rates of gastroenteritis caused by other bacterial pathogens including STEC, *Yersinia* and *L. monocytogenes* were extremely low, being 0.07, 0.03 and 0.01 per 100,000 population in 2020, respectively (European Food Safety Authority, 2021b).

A study conducted between 2012 and 2016 showed that *Campylobacter* was the leading enteric pathogen causing acute bacterial gastroenteritis in children in north-eastern urban and rural regions of Romania (Chiriac et al., 2017). Among the 615 cases involved in the study, 69.6% of cases (428/615) were caused by *Campylobacter*, followed by *Salmonella*(67/615), *E. coli*(29/615), *Shigella*(12/615) and *Y. enterocolitica*(3/615) (Chiriac et al., 2017).

##### 3.1.2.26 Slovakia

The rate of *Campylobacter* infection in Slovakia was reported to be high and on an upward trajectory. The reported incidence increased from 124.5 per 100,000 population in 2014, to 127.8 in 2017, and further rose to 141.1 in 2019 (**Table 1**) (European Centre for Disease Prevention and Control, 2019; European Food Safety Authority, 2021a). There has been a rapid decrease of *Campylobacter* infection in 2020, with the reported incidence being 90.2 per 100,000 population (European Food Safety Authority, 2021b). In addition to campylobacteriosis, Slovakia also had a high reported incidence of salmonellosis, namely 62.1 per 100,000 in 2020. The reported incidence rates of gastroenteritis caused by *Yersinia*, *L. monocytogenes* and STEC were much lower, being 3.1, 0.13 and 0.02 in 2020, respectively (European Food Safety Authority, 2021b).

##### 3.1.2.27 Slovenia

The highest reported incidence of campylobacteriosis in Slovenia was observed in 2016 (79.5 per 100,000 population). Since then, there has been a decreasing trend, the reported incidence dropped to 68.2 in 2017, 52.1 in 2019 and rapidly decreased to 38.7 in 2020 (**Table 1**). *Campylobacter* was the leading bacterial enteric pathogen causing gastroenteritis in Slovenia. The reported incidence of gastroenteritis caused by other bacterial pathogens including STEC, *L. monocytogenes*, *Yersinia*, and *Salmonella* varied between 1.02 and 1.4 in 2020 (European Food Safety Authority, 2021b).

##### 3.1.2.28 Spain

Although the reported incidence for campylobacteriosis has not been available in Spain since 2016, there has been a continuous increase in the number of cases notified during the past decade, from 11,481 cases in 2014, to 13,227 cases in 2015 and to 18,411 cases in 2018 (**Table 1**) (European Centre for Disease Prevention and Control, 2018a). In 2019 and 2020, 9,723 and 6,891 cases were reported, respectively. However, Spain did not receive data from all regions due to COVID-19 in 2019, therefore cases reported were lower than expected (European Food Safety Authority, 2021a). A recent study reported that in Madrid, Spain, the cases of *Campylobacter* infections reduced from 1,308 in 2019 to 391 in 2020, and *Salmonella* infections decreased from 462 in 2019 to 111 in 2020 due to social distancing and reduced tourism during the COVID-19 pandemic (De Miguel Buckley et al., 2020).

Ena et al. examined the epidemiology of patients with severe acute diarrhea between November 2016 and October 2018 (Ena et al., 2019). A total of 132 patients with acute diarrhea who required hospital admission were studied and *Campylobacter* was found to be the most frequently identified enteric pathogen (24/132), followed by *Clostridioides difficile*(20/132), *Salmonella*(20/132) and rotavirus (12/132) (Ena et al., 2019).

##### 3.1.2.29 Sweden

Sweden showed a conspicuous decreasing trend of campylobacteriosis during the period of 2016 to 2020, with the reported incidence dropping from 111.9 per 100,000 population in 2016, to 65.4 in 2019 and 33.3 in 2020 (**Table 1**) (European Food Safety Authority, 2021b). In 2019, a total of 6,693 cases were reported, among which 44% (2,865) cases were domestically acquired (National Veterinary Institute, 2020). A total of 137 isolates were collected from domestic cases, among which all but one were *C. jejuni*. The domestic incidence was higher among adults than children, and males (56%) had a higher incidence rate reported than females (National Veterinary Institute, 2020).

Publications on *Campylobacter* outbreaks in Sweden were rare. One *Campylobacter* outbreak was reported in 2014 in the South Western part of Sweden where 11 cases were identified (Lahti et al., 2017). *C. jejuni* was isolated from eight of the 11 cases examined and genomic analysis confirmed that human and cattle isolates of *C. jejuni* belonged to the same cluster, indicating cattle were the source of infection. The most likely route of transmission was found to be consumption of unpasteurised milk from the farm.

Gastroenteritis caused by other enteric pathogens including *Salmonella*, STEC, *Yersinia* and *L. monocytogenes* were less common, the reported incidence rates were 8.0, 4.8, 2.1 and 0.85 in 2020, respectively (European Food Safety Authority, 2021b).

##### 3.1.2.30 Switzerland

The trend of campylobacteriosis in Switzerland fluctuated in the years between 2014 and 2019, with the reported incidence ranging between 84.2 and 95.4 per 100,000 population (**Table 1**) (European Food Safety Authority, 2019a; European Food Safety Authority, 2021b). The reported incidence dropped to 71.7 in 2020. *Campylobacter* was the leading bacterial enteric pathogen causing gastroenteritis in Switzerland. The reported incidence of bacterial gastroenteritis caused by less prevalent pathogens including *Salmonella*, STEC and *L. monocytogenes* were 14.7, 8.4 and 0.67 per 100,000 population in 2020, respectively (European Food Safety Authority, 2021b). A study by Gosert et al. examined the detection of viral and bacterial pathogens from 677 stool samples collected from 504 patients with acute gastroenteritis between 2013 and 2015, and found that rotavirus (126/677), norovirus (82/677) and enterovirus (36/677) were the most prevalent viral pathogens, whilst *Clostridium difficile*(39/677) and *Campylobacter*(16/677) were the most prevalent bacterial pathogens (Gosert et al., 2018).

##### 3.1.2.31 United Kingdom

In 2019, a total of 58,718 cases of campylobacteriosis were reported in United Kingdom, with the reported incidence being 88.1 per 100,000 population, which was the lowest level in this country since 2014 (**Table 1**) (European Centre for Disease Prevention and Control, 2016; European Food Safety Authority, 2021a). Data for 2020 were not available in the European Union One Health 2020 Zoonoses Report due to the withdrawal of the United Kingdom from the European Union. *Campylobacter* was the leading bacterial enteric pathogen causing gastroenteritis in the United Kingdom, followed by *Salmonella*, STEC, *Yersinia* and *L. monocytogenes* whereby the reported incidence rates were 14.6, 2.4, 0.24 and 0.23 in 2019, respectively (European Food Safety Authority, 2021b).

Campylobacteriosis was the most commonly reported gastrointestinal infection in England and Wales with 56,729 cases reported in 2017 (Public Health England, 2018). The highest number of confirmed cases were found in the age group of 50-59 years old. Summer months including July and August were consistently shown to have the highest rate of infection (Public Health England, 2018). Hotels, schools, pubs and farms were settings where foodborne outbreaks frequently occur, and chicken liver containing dishes and raw drinking milks were causes for most of the foodborne outbreaks (Public Health England, 2018). During the Christmas period in 2016, a foodborne outbreak was reported in a hotel in North Yorkshire, England, of which 19 cases were identified, seven of them being positive for *Campylobacter*(Wensley et al., 2020). Chicken liver pâté was the food item that was most strongly associated with the disease, possibly due to inadequate cooking during the busy holiday period. In December 2016, an outbreak of campylobacteriosis was reported in North West England which affected 69 individuals. Further investigations found that the outbreak was associated with consumption of unpasteurised milk from a farm which was predominantly sold from a vending machine (Kenyon et al., 2020).

#### 3.1.3 Oceania

National surveillance of campylobacteriosis is available in Australia and New Zealand in Oceania.

##### 3.1.3.1 Australia

Data of campylobacteriosis in Australia were available for years 2014, 2015 and 2016, monitored by the National Notifiable Diseases Surveillance System and OzFoodNet.

There were 29,931, 22,573 and 24.164 cases of gastroenteritis caused by *Campylobacters* in Australia in 2014, 2015 and 2016, respectively (NNDSS Annual Report Working Group, 2019; Bell et al., 2021; NNDSS Annual Report Working Group, 2021). The reported incidence increased from 124.1 per 100,000 population in 2014 to 146.8 in 2016 (**Table 1**).

Although foodborne outbreaks were reported, *Campylobacter* infections in Australia were mainly sporadic. A total of 84 *Campylobacter* outbreaks were identified between 2001 and 2016 consisting of 1042 cases, among which 51 outbreaks were due to the consumption of contaminated food (Moffatt et al., 2020). Among foodborne outbreaks that had specific food vehicles identified (33/51), 85% (28/33) cases were due to chicken or chicken-containing dishes, and a significant higher proportion was due to poultry-liver containing dishes (11/28) (Moffatt et al., 2020). In addition to foodborne outbreaks, the remaining cases were caused *via* contaminated water (6%), transmission from animal to person (2.4%), person to person (3.6%), or unknown sources (27.4%) (Moffatt et al., 2020). Similar conclusions were drawn from a recent meta-analysis on sporadic campylobacteriosis in Australia and New Zealand between 1990 and 2017, in which consuming undercooked poultry and poultry cooked outside home, having pet chickens and overseas travel were considered relevant risk factors for sporadic campylobacteriosis (Varrone et al., 2020). Approximately 28% of *Campylobacter* infections in Australia were detected in summer, although they often occurred in other seasons as well. Males were more commonly affected than females with a male to female ratio of 1.2:1 (NNDSS Annual Report Working Group, 2019; Bell et al., 2021; NNDSS Annual Report Working Group, 2021)

The data of campylobacteriosis notification in New South Wales (NSW), a state of Australia, were available for the year 2018, with the reported incidence being 113.54 per 100,000 population (**Table 1**) (Communicable Diseases Branch, 2019).

*Campylobacter* was the leading bacterial enteric pathogen in Australia. In 2016, 49,885 notifications of gastrointestinal disease were reported, among which 24,164 were campylobacteriosis, followed by salmonellosis (18,088), shigellosis (1,406) and STEC (340) (NNDSS Annual Report Working Group, 2021). A recent study reported the isolation of enteric pathogens during 2015 and 2019 in a large diagnostic laboratory in NSW, reporting that the total positive isolates of *Campylobacters* from stool samples of patients with gastroenteritis during 2015 and 2019 was 11,597, followed by non-Typhi *Salmonella* and *Aeromonas* species, being 5,190 and 2,132, respectively (Yuwono et al., 2021).

##### 3.1.3.2 New Zealand

There has been a decreasing trend of *Campylobacter* infection in New Zealand between 2014 and 2019. The reported incidence decreased from 150.3 per 100,000 population in 2014, to 135.2 in 2017 and further dropped to 126.1 in 2019; the case numbers decreased from 6,776 in 2014, to 6,482 in 2017 and 6,202 in 2019 (**Table 1**) (The Institute of Environmental Science and Research Ltd, 2020; The Institute of Environmental Science and Research Ltd, 2021).

Only a minority of *Campylobacter* infections were due to outbreaks. In 2019, a total of 20 *Campylobacter* outbreaks were reported, amounting to 156 cases. The sources that caused *Campylobacter* outbreaks were not available, however, major risk factors causing campylobacteriosis were the consumption of contaminated food from retail premises, contacts with farm animals and consumption of contaminated water (Jeffs et al., 2019a). Similar risk factors were also identified in preceding years (The Institute of Environmental Science and Research Ltd, 2015; The Institute of Environmental Science and Research Ltd, 2016; The Institute of Environmental Science and Research Ltd, 2017; The Institute of Environmental Science and Research Ltd, 2019; The Institute of Environmental Science and Research Ltd, 2020).

Individuals in the age group of 0-4 years were found to have the highest reported incidence of *Campylobacter* infection compared to other age groups, being 242.7 per 100,000 individuals (**Table 1**). Furthermore, males (reported incidence of 146.7 per 100,000) were found to have a higher incidence rate than females (reported incidence of 106.1 per 100,000). There was a distinct seasonal pattern where the highest notification rate was found during early summer. This age, gender and seasonal distribution of campylobacteriosis was consistent with previous years except for 2016 where a large outbreak involving 964 cases occurred in Hawke’s Bay, contrary to the established summer peak pattern (The Institute of Environmental Science and Research Ltd, 2017). The outbreak was found to be due to the consumption of contaminated drinking water supplied by two bores on the outskirts of Havelock North (Institute of Environmental Science and Research Ltd, 2018).

*Campylobacter* was the leading bacterial enteric pathogen in New Zealand. The reported incidence of other enteric diseases including salmonellosis, yersiniosis, STEC infection and shigellosis in 2019 were 24.2, 24.1, 22.4 and 4.5 per 100,000 population, respectively, much lower than that of campylobacteriosis (The Institute of Environmental Science and Research Ltd, 2021).

*Campylobacter* is a major pathogen causing paediatric gastroenteritis in New Zealand. An observational study from 1997 to 2015 examining non-viral gastroenteritis in the paediatric population less than 15 years of age showed that *Campylobacter* was the most frequently notified pathogen, contributing to 51.7% of notified cases and 43.4% of hospitalisations (Jeffs et al., 2019b). Other major notified enteric pathogens included *Giardia*, *Cryptosporidium*, non-Typhi *Salmonella* and *Yersinia*. Most of the *Campylobacter* infections in New Zealand were caused by *C. jejuni*. Nohra et al. reported that between 2005 and 2014, among the 1,601 *Campylobacter* clinical isolates examined, 96% (1,552/1,601) were *C jejuni*, and only 2.9% (47/1,601) were *C. coli*(Nohra et al., 2016).

#### 3.1.4 North America

United States and Canada are the only two countries with national surveillance available for campylobacteriosis.

##### 3.1.4.1 Canada

Campylobacteriosis is monitored by The Public Health Agency of Canada’s FoodNet Canada surveillance system. The reported incidence of campylobacteriosis remained stable in Canada, ranging between 25.4 and 28.6 per 100,000 population in the years between 2014 and 2019 (**Table 1**) (Public Health Agency of Canada, 2018b; Public Health Agency of Canada, 2018a; Public Health Agency of Canada, 2019; Public Health Agency of Canada, 2021).

Species information was available for a subset of *Campylobacter* cases between 2017 and 2018. C*. jejuni* accounted for 88-91% of all cases examined, *C. coli* accounted for 5-7%, and the remaining 2-5% were caused by *C. upsaliensis*, *C. lari*, *C. fetus*, *C. rectus*, *C. curvus*, *C. hyointestinals*, or *C. ureolyticus*(Public Health Agency of Canada, 2018b; Public Health Agency of Canada, 2018a; Public Health Agency of Canada, 2019). Children aged between 1-5 years or late adolescents aged between 20-24 were found to have a high reported incidence rate, being more than 35 per 100,000 population. Males were consistently shown to be more easily infected than females. Higher proportions of *Campylobacter* cases were reported during summer months from June to August, with a marked decrease at the end of the summer season.

Although a higher number of cases were reported during summer months, proportions of retail chicken samples positive for *Campylobacter* continued to rise after summer. It was suggested that the summer peak might be due to improved survival and replication of bacteria under warm temperatures and seasonal changes in eating behaviours such as summer barbeques (Fleury et al., 2006; Public Health Agency of Canada, 2018b; Public Health Agency of Canada, 2018a; Public Health Agency of Canada, 2019).

A recent study examining potential bacterial enteric pathogens in children with acute gastroenteritis enrolled between December 2014 and February 2018 reported *Campylobacter*(18/2391) to be the third most prevalent bacterial pathogen, following *Salmonella*(54/2391) and *Aeromonas*(26/2391) (Tarr et al., 2019).

##### 3.1.4.2 United States

Campylobacteriosis in United States is monitored by the Foodborne Diseases Active Surveillance Network (Tack et al., 2020). The reported incidence of campylobacteriosis demonstrated an increasing trend for the period between 2014 and 2019, elevating from 13.3 per 100,000 population in 2014, to 19.2 in 2017, and 19.5 in 2019 (**Table 1**) (Centers for Disease Control and Prevention, 2014; Centers for Disease Control and Prevention, 2017; Marder et al., 2017; Marder et al., 2018; Tack et al., 2019; Tack et al., 2020). There was a decrease in the rate of *Campylobacter* infection in 2020, in which the reported incidence dropped to 14.4 per 100,000 population (Ray et al., 2021).

According to the FoodNet Surveillance reports for 2014 and 2015 where species information was available for subsets of *Campylobacter* infections, 88% of cases were caused by *C. jejuni*, 7-8% of cases were caused by *C. coli* and the rest by other *Campylobacter* species (Centers for Disease Control and Prevention, 2014; Centers for Disease Control and Prevention, 2017). Children less than five years old had the highest reported incidence (22.2 per 100,000 population for 2014 and 20.8 for 2015) compared to other age groups. Males were found to be more frequently infected than females. The summer months of June, July and August were found to have the highest number of infections.

Although several *Campylobacter* outbreaks associated with a variety of sources have been reported in United States in recent years, *Campylobacter* infections in United States were mainly sporadic. A *C. jejuni* outbreak linked to puppy exposure affecting 118 individuals was reported in Ohio between 2016-2018 (Montgomery et al., 2018). A cluster of *C. jejuni* infection involving 39 cases in Nebraska was reported in 2017 which was found to be associated with contaminated municipal water supply (Pedati et al., 2019). *C. jejuni* outbreaks due to the consumption of raw milk were reported in Colorado in 2016, and Utah in 2014, which affected 99 and 17 persons, respectively (Davis et al., 2016; Burakoff et al., 2018). Lanier et al. have reviewed 28 chicken liver-associated outbreaks of campylobacteriosis and salmonellosis in United States between 2000 and 2016. Among the 28 outbreaks, 23 were campylobacteriosis, three were salmonellosis and two were caused by both bacterial pathogens (Lanier et al., 2018). Common features of these outbreaks included chicken liver dishes, inadequate cooking and food preparation settings (Lanier et al., 2018). Overall, contacts with animals, drinking raw milk, and the consumption of contaminated food, especially chicken dishes, were the major causes of *Campylobacter* outbreaks in United States.

*Campylobacter* was the leading foodborne bacterial pathogen causing gastroenteritis in United States. In 2019, a total of 25,866 foodborne infections were reported, among which 9,731 cases were caused by *Campylobacter*, followed by *Salmonella*(8,556 cases), STEC (3,127), *Shigella*(2,416), *Yersinia*(681), *Vibrio*(466) and *Listeria*(134) (Tack et al., 2020). Among the 9,731 *Campylobacter* infections, 85% were acquired domestically (Tack et al., 2020).

### 3.2 Campylobacteriosis in countries without national surveillance data

#### 3.2.1 Africa

Currently there are no national surveillance data available in Africa. However, several studies have examined the enteric pathogens in this region.

##### 3.2.1.1 Madagascar, Malawi and South Sudan

In Africa, *Campylobacter* was not the leading bacterial enteric pathogen causing gastroenteritis (**Table 2**). In Madagascar, EPEC (3%, 6/199) was found to be the leading bacterial enteric pathogen causing diarrhea in children less than five years old, followed by *Shigella*(1.5%, 3/199) and *Campylobacter*(1%, 2/199) (Randremanana et al., 2016). Similarly, in Malawi, enteroaggregative *E. coli*, heat-stable enterotoxin-producing *E. coli* and EPEC were the leading bacterial enteric pathogens among children hospitalised with diarrhea, accounting for 51.8% (354/684), 21.2% (145/684) and 18% (123/684) of all cases, respectively. Consistently, Biswas et al. in 2017 reported diarrheagenic *E. coli*(68.3%) to be the major pathogen of diarrhea in the United Kingdom military personnel in South Sudan, followed by *Salmonella*(50.1%), and *Campylobacter*(0.8%, 10/127) (Biswas et al., 2019).

##### 3.2.1.2 Ethiopia and Ghana

The presence of *Campylobacter* in HIV patients with diarrhea was reported by Ayele1 et al. in Ethiopia during 2019, in which *Campylobacter*, although being the most predominant enteric bacterial pathogen, had a low isolation rate of 4.4% (8/180), followed by *Salmonella*(2.8%, 5/180) and *Shigella*(1.1%, 2/180) (Ayele et al., 2020). Forson et al. assessed the incidence of *Campylobacter* in HIV patients with gastroenteritis in Ghana between 2015 and 2016, and reported that 50.7% (71/140) were positive for *Campylobacter*; surprisingly all isolates were *C. coli*(Forson et al., 2020).

##### 3.2.1.3 Egypt

Studies from Egypt reported *Campylobacter* detection rates from 4.1-48% in patients with gastroenteritis (**Table 2**).

A study by Zeinhom et al. in 2019 reported a *C. jejuni* isolation rate of 48% (12/25) from individuals with diarrhea across Beni-Suef Governorate; however, the detection rate was only slightly higher than that from non-diarrheic individuals (32.1%, 9/28) (Zeinhom et al., 2021). Barakat et al. reported that 38.1% (40/105) of diarrheic human stools collected in 2018 from five different governorates were PCR positive for *Campylobacter*, among which a majority of them were *C. jejuni*(92.5%, 37/40) (Barakat et al., 2020). A similar detection rate of *C. jejuni* was reported in Sharkia (30%, 30/100) and Ismailia (31.2%, 25/80) Governorates between 2017-2018 (Ammar et al., 2020; Abdelmageed et al., 2021). However, a much lower detection rate of *C. jejuni* was reported in multiple Governorates between 2015 and 2016 (17.33%, 13/75), as well as between 2013-2014 (21.5%, 20/93) (Elgabry et al., 2016; Ghoneim et al., 2020).

Among all the Egyptian regions that have *Campylobacter* infection reported, Zagazig city had the lowest isolation rate of *C. jejuni*. The study by Ahmed et al. examining the presence of *C. jejuni* from 270 stool samples collected from gastroenteritis patients between 2015-2018 showed that only 4.1% (11/270) were positive for *C. jejuni*(Abd El-Hamid et al., 2019). A very similar detection rate of *C. jejuni* was reported between 2013-2014 (4.1%, 10/246) (Ahmed et al., 2015).

Taken together, there seems to be an increasing trend of *Campylobacter* infection in Egypt, however the lack of national surveillance makes this difficult to conclude.

#### 3.2.2 Asia

##### 3.2.2.1 Bangladesh

Two recent studies examining the incidence of *Campylobacter* in diarrheal patients between 2019-2020 in Dhaka and Mymensingh reported very different results, possibly due to the geographical differences where the studies were conducted (**Table 2**). It was found that in Dhaka, *Campylobacter* was the third leading bacterial pathogen being isolated from diarrheal stool samples (3.6%, 76/2135), following *V. cholera*(23.8%, 509/2135) and *Aeromonas*(17%, 363/2135) (Garbern et al., 2021). On the other hand, a much higher isolation rate was reported in Mymensingh in which 31.5% (104/330) of clinical samples had *Campylobacter*. Of these, 21.8% (72/330) were *C. jejuni* and 9.6% (32/330) were *C. coli*(Rahman et al., 2021). Additionally, a higher detection rate of *Campylobacter* was found in children less than five years old, contributing to more than half of all positive cases (Rahman et al., 2021).

##### 3.2.2.2 China

*Campylobacter* infections have been reported in multiple regions of China (**Table 2**). The most recent study examining the presence of *Campylobacter* in diarrheal patients between 2017-2019 in Wenzhou showed that 10.5% of patient stool samples had *Campylobacter* species isolated; this being the highest detection rate reported in China in the past 15 years (Zhang et al., 2020a). Li et al. examined the presence of *Campylobacter* in 370 diarrhea cases between 2016-2017 in Beijing. *Campylobacter* was the third leading enteric bacterial pathogen found in 7% (26/370) of total cases, following diarrheagenic *E. coli*(7.3%, 27/370) and *V. parahaemolyticus*(10.3%, 38/370); other enteric bacterial pathogens included *Salmonella*(6.2%, 23/370) and *Shigella*(0.3%, 1/370) (Li et al., 2018). A similar *Campylobacter* detection rate of 7.8% between 2017-2018 was reported by Zhang et al. (Zhang et al., 2020b).

A study on the aetiology of acute diarrhea among children under five years of age in Wuhan between 2014-2015 reported that rotavirus (25.7%, 98/381) was the leading viral enteric pathogen, whilst *Salmonella*(8.4%, 32/381), diarrheagenic *E. coli*(4.7%, 18/381) and *Campylobacter*(2.9%, 11/381) were the most prevalent bacterial pathogens (Zhu et al., 2016).

Another study by Wang et al. reported a very low isolation rate of *Campylobacter*(0.13%, 1/755) in children less than five years old in a developing region (Henan Province) between 2011 and 2014, while none of the patients (0/1422) from the developed region (Beijing) were found to be positive for *Campylobacter*(Wang et al., 2015). Other pathogens detected in the developing region of Henan included *Shigella*(16.95%, 128/755), *Salmonella*(5.3%, 40/755), diarrheagenic *E. coli*(7.15%, 54/755), *Aeromonas hydrophila*(3.58%, 27/755) and *Yersinia*(0.26%, 2/755) (Wang et al., 2015).

A large scale national-based prospective surveillance study on more than 90,000 patients with acute diarrhea across 31 provinces in China was conducted between 2009-2018. It was found that rotavirus A and norovirus were the two leading viral enteric pathogens, while *C. jejuni* was the sixth leading bacterial enteric pathogen detected (0.87%) following diarrheagenic *E. coli*(6.71%), non-Typhi *Salmonella*(4.41%), *Shigella*(2.44%), *V. parahaemolyticus*(2.08%) and *A. hydrophila*(0.99%) (Wang et al., 2021).

Few studies have reported *Campylobacter* outbreaks in China. In August 2019, a gastroenteritis outbreak consisting of 14 patients working at the same factory was reported in Shunyi District Beijing. The diarrhea happened after lunch which was supplied from a meal delivery company. Further examinations showed that 12 of the 14 patients, as well as two food workers were PCR positive for *C. jejuni*(Li et al., 2020b). Further investigations by a later study on the same outbreak did not identify the original source of infection, however duck blood curd was considered as a possible risk factor (Chen et al., 2021). Two studies conducted in 2018 reported a *Campylobacter* outbreak in students and teachers in Beijing following a school trip and in Hangzhou due to unknown sources (Qu et al., 2019; Yu et al., 2020). *C. jejuni* was found to be the causative agent of both outbreaks.

##### 3.2.2.3 India

Limited data are available on *Campylobacter* infection in India (**Table 2**). A recent study examining 400 human dysenteric stool samples between 2019 and 2020 in Vellore, South India using PCR analysis showed that EPEC (17%, 68/400) was the most dominant bacterial enteric pathogen being detected, followed by *Campylobacter*(12%, 48/400) and EIEC/*Shigella*(6.5%, 26/400) (Lakshmi Ss et al., 2022). By using genus specific PCR methods, a study by Mohakud et al. detected *Campylobacter* in 16.77% (52/310) of faecal samples collected from children with acute diarrhea in the peri-urban Bhubaneswar city between 2016-2017 (Mohakud et al., 2019). Infants between two and five years old were shown to have the highest rate of *Campylobacter* positivity (23.3%, 14/60). Co-infection with other enteric pathogens such as rotavirus, *E. coli*, *Shigella*, *Cryptosporidium* and adenovirus was also observed (Mohakud et al., 2019). Another study examining the presence of *Campylobacter* enteritis in children under five years in Northeast India between 2014-2015 reported a detection rate of 10.1% (41/407), of which 8.1% (33/407) was *C. jejuni*(Borkakoty et al., 2020). The highest detection rate was reported during summer (June and July, 20-18.8%) (Borkakoty et al., 2020).

##### 3.2.2.4 Iran

A recent study by Alsalmi et al. showed that in 2019, *C. jejuni*(0.6%, 5/790) was the second most dominant enteric bacterial pathogen causing gastrointestinal manifestations in Oman, following *Salmonella*(2.5%, 20/790) (**Table 2**) (Alsalmi et al., 2021). The detection rates of *C. jejuni* in diarrheal patients from Mazandaran and East Azerbaijan in 2017 and 2016 were reported to be 27% (20/74) and 27.8% (62/223), respectively (Ranjbar et al., 2017; Divsalar et al., 2019).

Mazaheri et al. reported that in Semnan between 2014 and 2015, *Campylobacter* was isolated from stools of 8.6% (36/419) of children (1-12 years old) with diarrhea; children in age group 6-12 years were found to have the highest frequency of positive cultures (Mazaheri et al., 2016). Around the same time between 2013-2014 in Hamadan, 10% (12/120) of the diarrheal stool samples from children less than 10 years old had *Campylobacter* isolated. In Central Iran, 33% (76/230) diarrheal stools from children were PCR positive for *C. jejuni* in 2015, among which most of the infections occurred in the age group of 1-3 years (Rastyani et al., 2015; Abbasi et al., 2019). *Campylobacter* infection was found to be more prevalent in girls in Semnan, contrary to that of Central Iran (Mazaheri et al., 2016; Abbasi et al., 2019).

In summary, by reviewing the incidence studies on *Campylobacter* infection in Iran, the positive detection rates in different regions ranged from 3.6% to 35.4%; campylobacteriosis was frequently reported in children with diarrhea (**Table 2**).

##### 3.2.2.5 Iraq

In Iraq, during 2017, adenovirus (34.2%, 53/155) was the leading enteric pathogen causing acute diarrhea in children less than five years old, followed by *Salmonella*(14.8%, 23/155), *Entamoeba*(13.5%, 21/155) and *Campylobacter*(10.9%, 17/155) (Harb et al., 2019).

##### 3.2.2.6 Lebanon

The isolation rate of *Campylobacter* was reported to be 12% (35/291) in Lebanon in 2018 in children under 12 years with acute gastroenteritis (Ghssein et al., 2021). A large-scale incidence study including 1000 patients with diarrhea performed between 2016-2017 in Lebanon reported *Campylobacter* as the leading bacterial enteric pathogen with a detection rate of 21.5%. Other pathogens being detected were rotavirus (6.7%), *Entamoeba histolytica*(6%), *Salmonella* Typhi (5.8%), and enterohemorrhagic *E. coli* O157:H7 (5.6%) (Ibrahim et al., 2019). Males were found to have a slightly higher *Campylobacter* isolation rate as compared to females; there was a clear seasonal distribution, with summer having the highest rate of infection (Ibrahim et al., 2019).

##### 3.2.2.7 Nepal

*Campylobacter* infection in children less than five years old with acute gastroenteritis in Kathmandu during 2017-2018 was reported (**Table 2**) (Bhattarai et al., 2020). Among the 303 children participating, more than half (56.8%, 172/303) were infected with *Campylobacter*, of which 26.7% were *Campylobacter* mono-infection, and 30% were co-infected with rotavirus. Children less than six months were found to have an increased risk of *Campylobacter* mono-infection as compared to other age groups (Bhattarai et al., 2020).

A case-control study of traveller’s diarrhea was conducted in Kathmandu between 2012-2014 in adults from North America, Western Europe, Australia, New Zealand, Japan, Taiwan or Korea residing in Nepal for less than one year (Murphy et al., 2019). *Campylobacter* was the second leading bacterial enteric pathogen (20%, 88/433), following enteroaggregative *E. coli*(26%, 110/420) (Murphy et al., 2019). Another epidemiological surveillance study of traveller’s diarrhea conducted between 2011 and 2014 for residents of aforementioned countries visiting Nepal reported a significantly higher presence of *C. concisus* in traveller’s diarrhea cases (28.9%, 24/83) as compared with asymptomatic controls (4%, 3/75), suggesting emerging *Campylobacter* species also contributed to the traveller’s diarrhea in this area (Serichantalergs et al., 2017).

##### 3.2.2.8 Pakistan

Noreen et al. reported that in 150 diarrheal stool samples collected from hospitalised paediatric patients between December 2014 and September 2015 in major cities of Pakistan, *C. jejuni* was isolated in 54.6% (82/150) of them (**Table 2**) (Noreen et al., 2020). This isolation rate was similar to that reported in Rawalpindi and Islamabad in 2014 in which 52% (260/500) of paediatric diarrheal stool samples were culture positive for *Campylobacter*. *C. jejuni* accounted for 48.2% (241/500) of all cases, and the positive rate was much higher than that of rotavirus (26.4%) (Sadiq et al., 2019b).

##### 3.2.2.9 Thailand

Analogous to Nepal, traveller’s diarrhea was often reported in Thailand, which was also a prevalent illness encountered by deployed military personnel (**Table 2**). A prospective study of acute diarrhea in United States military personnel on deployment in Thailand between 2013-2017 reported *Campylobacter* as the leading bacterial enteric pathogen, causing 43.8% (21/48) of all cases, followed by diarrheagenic *E. coli*(42%, 20/48), and *Salmonella*(23%, 11/48) (Lurchachaiwong et al., 2020). Another study by Lertsethtakarn et al. also found that *Campylobacter*(31.2% 48/154) was the most prevalent enteric pathogen causing traveller’s diarrhea for individuals visiting Thailand from North America, Europe, Australia, New Zealand, Japan, Taiwan, and South Korea between 2012-2014 (Lertsethtakarn et al., 2018). Additionally, non-jejuni/coli *Campylobacter* species such as *C. concisus* and *C. ureolyticus* were found to be causative agents for 11.5% (3/26) and 7.7% (2/26) of traveller’s diarrhea, respectively between 2011-2014 (Serichantalergs et al., 2017).

##### 3.2.2.10 United Arab Emirates

In the United Arab Emirates between 2017-2019, *Campylobacter* only contributed to a minor portion (1.9%, 4/203) of diarrheal cases in children less than five years of age, with the leading viral enteric pathogens being rotavirus (21.2%, 43/203) and norovirus (19.2%, 39/202), and leading bacterial species being EPEC(17.7%, 36/203) and enteroaggregative *E. coli*(10.3%, 21/203) (Alsuwaidi et al., 2021).

#### 3.2.3 Europe

##### 3.2.3.1 North Macedonia

Trajkovska-Dokic et al. reported that the presence of *Campylobacter* infection in patients with acute enteritis between 2012-2017 was 2.5% (97/3820), among which 98.63% were *C. jejuni*(85/97) and 12.37% (12/97) were *C. coli*(**Table 2**) (Trajkovska-Dokic et al., 2019). There was an increase in the *Campylobacter* isolation rate in 2016 (3.8%) and 2017 (6.4%) as compared with previous years, where the rates were 1.4% or below (Trajkovska-Dokic et al., 2019). This study also found a significantly higher isolation rate in individuals under 15 years old as compared those from 15-30, 31- 50 or above, but no statistical difference between genders was noted

#### 3.2.4 Oceania

##### 3.2.4.1 Papua New Guinea

Between 2013 and 2014, a study on acute diarrhea was conducted in four provinces in Papua New Guinea including Hela, Eastern Highlands, Madang, and Central Provinces (**Table 2**). The common enteric pathogens detected were *Shigella*(38.1% 45/118), *Campylobacter*(33.1%) and rotavirus (20.3%) (Abdad et al., 2020).

### 3.3 Human hosted *Campylobacter* species

Some of the *Campylobacter* species utilise humans as their natural host and have not been isolated from other animals, which were referred to as human hosted *Campylobacter* species (Liu et al., 2018). Among the human hosted *Campylobacter* species, *C. concisus* was most studied. *C. concisus* normally colonises the human oral cavity as a commensal oral bacterium (Zhang, 2015). The presence of *C. concisus* in the lower parts of the human gastrointestinal tract is associated with gastrointestinal diseases such as Barrett’s oesophagus, gastroenteritis and inflammatory bowel disease (Macfarlane et al., 2007; Yang et al., 2009; Zhang et al., 2009; Man et al., 2010; Mahendran et al., 2011; Mukhopadhya et al., 2011; Zhang, 2015; Kirk et al., 2016; Liu et al., 2018). *C. concisus* has two genomospecies (Chung et al., 2016). Strains of genomospecies 2 C*. concisus* are more likely to colonise the human gastrointestinal tract (Engberg et al., 2005; Kalischuk and Inglis, 2011; Ismail et al., 2012; Wang et al., 2017). *C. concisus* is able to induce gut barrier defects and intestinal inflammation (Ismail et al., 2012; Mahendran et al., 2016). Additionally, a recent study showed that *C. concisus* has a immunomodulatory role by upregulating programmed death-ligand 1 in interferon-γ sensitised intestinal epithelial cells (Lee et al., 2021). Details on disease associations and pathogenic mechanisms of human hosted *Campylobacter* species were reviewed recently (Liu et al., 2018).

### 3.4 The impact of COVID-19 on *Campylobacter* infection

The epidemiological data of campylobacteriosis for the year 2020 were available in 27 countries, including 26 European countries and the United States, providing the opportunity for assessing the impact of COVID-19 on campylobacteriosis. The average incidence of campylobacteriosis reported during the years 2014-2019 was compared to that in 2020, the first pandemic year of COVID-19. There has been a drop in the reported incidence of campylobacteriosis in 22 of the 26 European countries, except France, Latvia, Luxembourg and Lithuania (**Table 1**; **Supplementary Figure 1**).

In the United States, the decreased incidence reported was observed not only for campylobacteriosis but also enteric infections caused by other bacterial pathogens (Ray et al., 2021). A study from the United States showed that in 2020, the reported incidence of infections caused by enteric pathogens dropped by 26% as compared with 2017-2019, with only 18,462 cases reported. Furthermore, only 5% (958) of infections were associated with international travel as compared to 14% during 2017-2019, and most of these (83%, 798/958) cases occurred during January-March.

## 4 Discussion and conclusion

Globally, national surveillance data of campylobacteriosis were available from 38 countries, mostly from countries in Europe (30 countries) (**Table 1**). The other countries with a national surveillance program for campylobacteriosis were United States, Canada, United Kingdom, Australia, New Zealand, Japan, Singapore and Korea.

**Figure 1:**
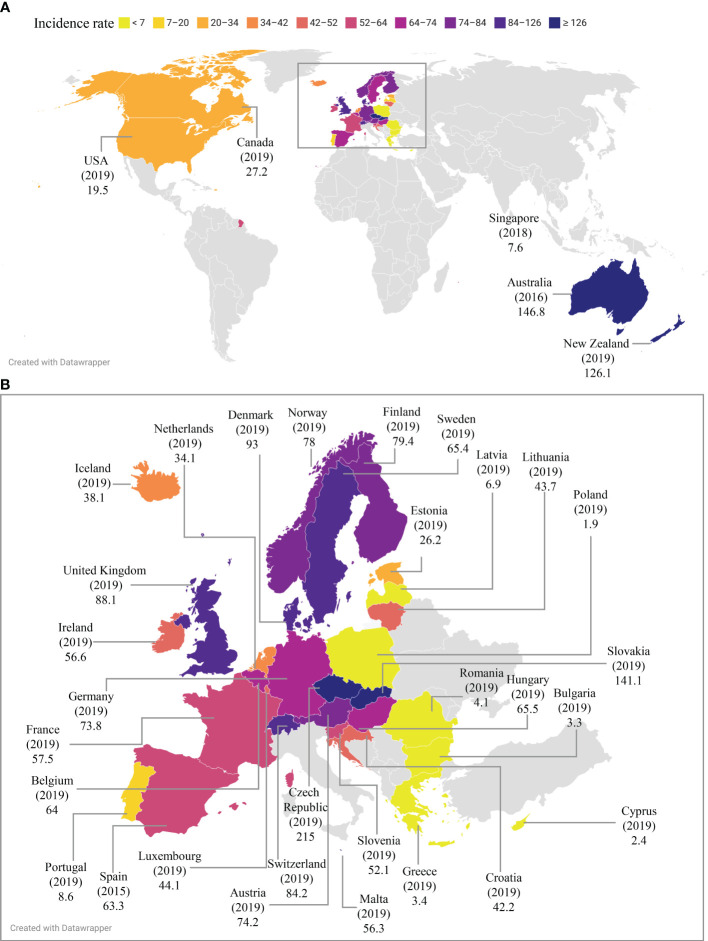
Reported global incidence of campylobacteriosis. Reported incidence rates of campylobacteriosis worldwide **(A)** and in Europe **(B)** are presented as per 100,000 population. Data presented in the figure were mainly from 2019. In countries where the 2019 data were not available, data from a previously available year were used. Countries with no reported incidence of campylobacteriosis available were in grey colour. Map was created with Datawrapper.

The reported incidence of campylobacteriosis in countries with a national surveillance program varied greatly (**Figure 1**; **Table 1**). Czech Republic in eastern Europe had the highest reported incidence of campylobacteriosis worldwide followed by Australia and New Zealand. Some other European countries such as Bulgaria, Poland and Romania had a low reported incidence rate of campylobacteriosis. Countries in western and northern Europe in general had a high reported incidence of campylobacteriosis (**Figure 1**; **Table 1**). Limited data were available from countries in southern Europe. The reported incidence of campylobacteriosis in the United States and Canada was relatively low amongst developed countries (**Figure 1**; **Table 1**). *Campylobacter* was the leading bacterial enteric pathogen in these countries.

Campylobacteriosis surveillance programs provide an excellent opportunity in obtaining national data. However, the data accuracy depends on the implementation of such programs in individual countries. Due to the differences in medical and reporting systems, it is not surprising that cases of campylobacteriosis may be underreported in some countries. This, at least in part, would have contributed to their low reported campylobacteriosis incidence. Campylobacteriosis incidence may vary in different regions of a given country, particularly in large countries comprising of different climate, economic and cultural zones. The regional differences were not revealed in national data presented in this review. Despite these limitations, data obtained from national surveillance programs still provide very useful information in assessing epidemiology trend of campylobacteriosis. Individual studies reporting *Campylobacter* infections in humans during 2014-2021 in each country, if available, were also included in this review, which provided further information on global *Campylobacter* infection. However, in countries that lacked campylobacteriosis surveillance programs, these individual studies do not represent the overall epidemiology of their nations, due to the restricted subset of population that these studies were performed on.

Culture-independent molecular method is more sensitive than bacterial isolation in detecting *Campylobacter* species in clinical samples. In European countries, the diagnosis was generally based on cultures from human faecal samples and confirmed by both culture and PCR methods (European Food Safety Authority, 2021b). In the United States, culture-independent diagnostic test was used (Ray et al., 2021). In Canada, confirmed cases were defined as positive isolation of *Campylobacter* from an appropriate clinical specimen (Government of Canada, 2018). Furthermore, different detection methods were used in individual studies. The diagnostic methods used for *Campylobacter* detection could also contribute to variations in *Campylobacter* detection rates between different countries and studies.

Consumption of contaminated chicken containing food seems to be the most common driver of *Campylobacter* infection in developed countries. For example, in Europe, occurrence of *Campylobacter* in poultry meat is an important vehicle for *Campylobacter* infection (Guirin et al., 2020). In Australia and New Zealand, consumption of undercooked chicken or chicken-containing dishes, consumption of contaminated water, owning pet chicken and travelling overseas are major risk factors of *Campylobacter* infection (Moffatt et al., 2020; Varrone et al., 2020). In Canada, consumption and handling of contaminated poultry meat appeared to be the most common drivers of human campylobacteriosis (Huang et al., 2015). In United States, *Campylobacter* outbreaks were frequently attributed to dairy products, chicken, and vegetables (Sher et al., 2021). While outbreaks were more frequently reported in some countries such as Japan, *Campylobacter* infections were mainly sporadic cases in most of the developed countries. This difference may reflect cultural differences in lifestyle.

Although three Asian countries had national surveillance data of campylobacteriosis, the reported incidence was only available in Singapore (**Figure 1**; **Table 1**). Data in other Asian countries included in this review were mainly from individual studies on isolation and detection of enteric pathogens. In some Asian countries such as Lebanon, Japan and Thailand, *Campylobacter* was the leading bacterial enteric pathogen. In other Asian countries such as Korea, Singapore, Iran and Iraq, *Campylobacter* was the second or third leading bacterial enteric pathogen (Ministry of Health Singapore, 2017; Harb et al., 2019; Alsalmi et al., 2021; Korea Disease Control and Prevention Agency, 2021); *E. coli*, *Salmonella* or *Vibrio* species was the leading bacterial enteric pathogens in these countries. Although national data are not available from China, a large scale national-based prospective surveillance study showed that *C. jejuni* was the sixth leading bacterial enteric pathogen (Wang et al., 2021). *Campylobacter* was reported to be the third leading bacterial enteric pathogen in several countries in Africa, following *E. coli* and *Salmonella* species. These data show that *Campylobacter* infection is also an important health concern in Asian countries.

*C. jejuni* is the major causative species of campylobacteriosis, responsible for about 90% of cases. *C. coli* accounted for less than 10% of *Campylobacter* caused gastrointestinal infections (Ministry of Health Singapore, 2019; Public Health Agency of Canada, 2019; European Food Safety Authority, 2021b). Other *Campylobacter* species such as *C. upsaliensis*, *C. lari*, *C. fetus*, *C. rectus*, *C. curvus*, *C. hyointestinals*, *C. ureolyticus* and *C. concisus* were also reported, although they contributed to a small portion of *Campylobacter* infections. *Campylobacter* may also co-infect humans with other bacterial enteric pathogens (Yuwono et al., 2021)

The seasonality of campylobacteriosis varied in different countries. *Campylobacter* infection was more common in the summer period in some countries such as European countries, Japan, Korea, Canada and United States (**Table 1**). A winter peak was also observed during the Christmas festive season in some countries in Europe (Bless et al., 2017). In a recent study from New South Wales of Australia, *Campylobacter* infection did not display seasonality (Yuwono et al., 2021). *Campylobacter* infection was more common in males than females. These findings show that multiple factors contribute to the risk of developing *Campylobacter* infections.

The reported incidence and case numbers of campylobacteriosis have remained steadily high in most developed countries prior to the COVID-19 pandemic, although with fluctuations between years (**Table 1**). It showed an increasing trend in some countries such as France and Japan. The COVID-19 pandemic has reduced the reported incidence of campylobacteriosis in most of the countries where 2020 epidemiology data were available (**Table 1**; **Supplementary Figure 1**). In response to the COVID-19 pandemic, many countries have implemented specific policies such as restricting social gathering, restricting travel, closing restaurants and schools. These interventions have reduced the exposure of humans to foodborne pathogens, consequently reducing the infections caused by *Campylobacter* species and other enteric pathogens (European Food Safety Authority, 2021b; Ray et al., 2021). Furthermore, during the COVID-19 pandemic, reduced access to medical diagnostics was also likely to contribute to the reduction of campylobacteriosis. It is expected that *Campylobacter* infections will increase post COVID-19 pandemic as normal ways of life resume.

In conclusion, *Campylobacter* species is one of the most common human enteric pathogens in both developed and developing countries, with campylobacteriosis remaining a global health concern. Increased research and improved strategies are needed for the prevention and reduction of *Campylobacter* infection.

## Author contributions

LZ conceived the project. FL and LZ wrote the first version of the manuscript. SL, JX, and SR provided critical feedback and helped with reviewing and editing the manuscript. All authors contributed to the article and approved the submitted version.

## Funding

This work is supported by a Faculty Research Grant awarded to LZ from the University of New South Wales (grant number PS46772).

## Conflict of interest

The authors declare that the research was conducted in the absence of any commercial or financial relationships that could be construed as a potential conflict of interest.

## Publisher’s note

All claims expressed in this article are solely those of the authors and do not necessarily represent those of their affiliated organizations, or those of the publisher, the editors and the reviewers. Any product that may be evaluated in this article, or claim that may be made by its manufacturer, is not guaranteed or endorsed by the publisher.

## Supplementary material

Supplementary Figure 1 Impact of COVID-19 on the reported incidence of campylobacteriosis. Reported incidence are presented as per 100,000 population. The average incidence between 2014-2019 was lower than that in 2020 due to COVID-19 pandemic in most of the countries, except France, Lithuania and Malta.
