# Supplementary figures and images for: Global epidemiology of campylobacteriosis and the impact of COVID-19
Source: Front Cell Infect Microbiol. 2022 Nov 28;12:979055. doi: 10.3389/fcimb.2022.979055 (PMC9742372; doi:10.3389/fcimb.2022.979055)

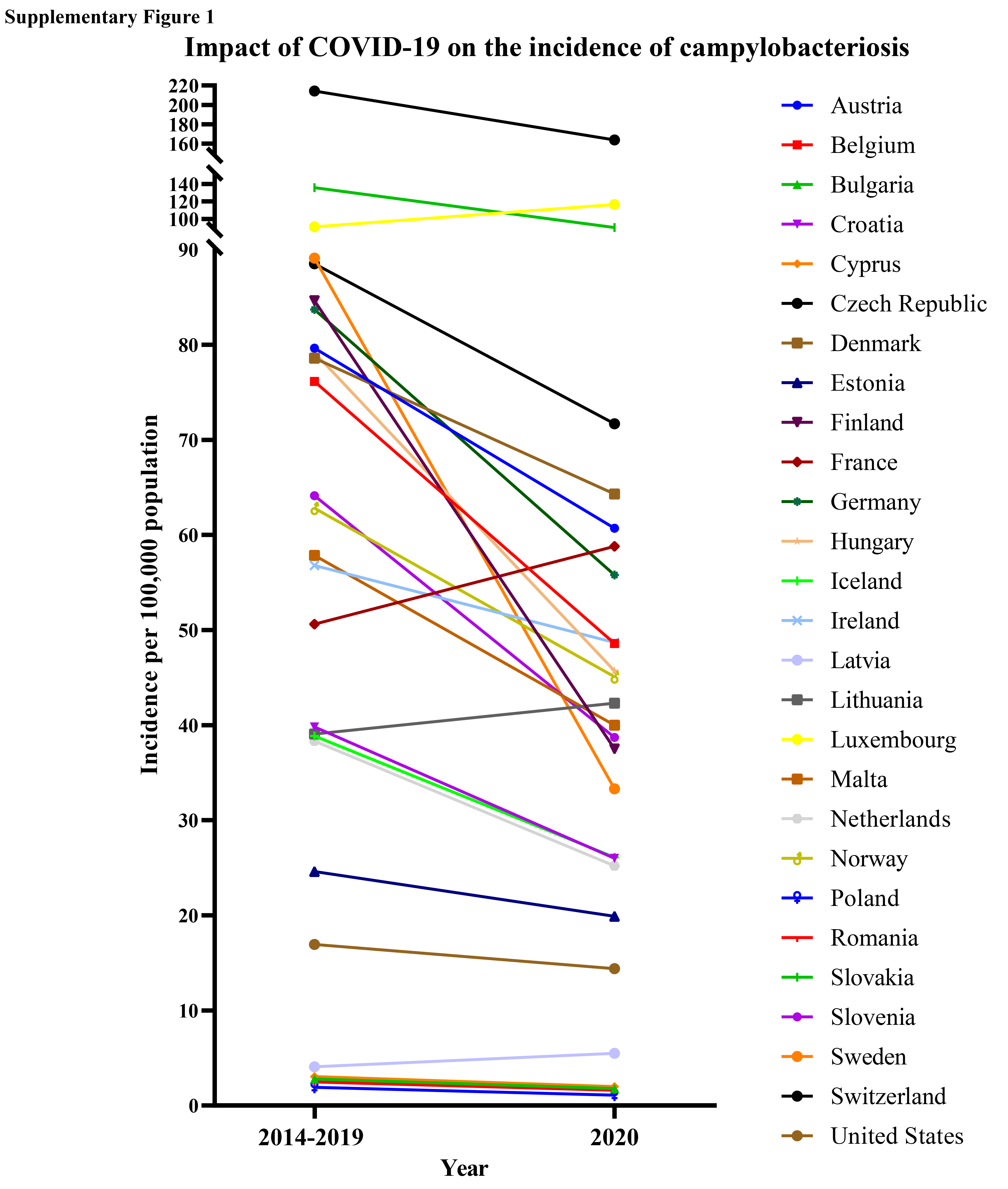

Supplement: Supplementary Figure 1 — Impact of COVID-19 on the reported incidence of campylobacteriosis. Reported incidence are presented as per 100,000 population. The average incidence between 2014-2019 was lower than that in 2020 due to COVID-19 pandemic in most of the countries, except France, Lithuania and Malta. [file Image_1.tif]
